# High-efficiency targeted transgene integration via primed micro-homologues

**DOI:** 10.1038/s41421-023-00552-0

**Published:** 2023-07-04

**Authors:** Chenxin Wang, Sen Fang, Yangcan Chen, Na Tang, Guanyi Jiao, Yanping Hu, Jing Li, Qingtong Shan, Xin Wang, Guihai Feng, Qi Zhou, Wei Li

**Affiliations:** 1 State Key Laboratory of Stem Cell and Reproductive Biology, Institute of Zoology, Chinese Academy of Sciences, Beijing, China; 2 Institute for Stem Cell and Regenerative Medicine, Chinese Academy of Sciences, Beijing, China; 3 University of Chinese Academy of Sciences, Beijing, China; 4 Bejing Institute for Stem Cell and Regenerative Medicine, Beijing, China; 5 Northeast Agricultural University, Harbin, China

**Keywords:** Double-strand DNA breaks, DNA recombination

## Abstract

Due to the difficulties in precisely manipulating DNA repair pathways, high-fidelity targeted integration of large transgenes triggered by double-strand breaks is inherently inefficient. Here, we exploit prime editors to devise a robust knock-in (KI) strategy named primed micro-homologues-assisted integration (PAINT), which utilizes reverse-transcribed single-stranded micro-homologues to boost targeted KIs in different types of cells. The improved version of PAINT, designated PAINT 3.0, maximizes editing efficiency and minimizes off-target integration, especially in dealing with scarless in-frame KIs. Using PAINT 3.0, we target a reporter transgene into housekeeping genes with editing efficiencies up to 80%, more than 10-fold higher than the traditional homology-directed repair method. Moreover, the use of PAINT 3.0 to insert a 2.5-kb transgene achieves up to 85% KI frequency at several therapeutically relevant genomic loci, suggesting its potential for clinical applications. Finally, PAINT 3.0 enables high-efficiency non-viral genome targeting in primary T cells and produces functional CAR-T cells with specific tumor-killing ability. Thus, we establish that the PAINT method is a powerful gene editing tool for large transgene integrations and may open new avenues for cell and gene therapies and genome writing technologies.

## Introduction

CRISPR/Cas9-based gene editing tools achieve targeted integration of large foreign DNA fragments mainly by exploiting endogenous DNA repair pathways, such as homology-directed repair (HDR), non-homologous end joining (NHEJ), and an alternative NHEJ repair pathway named microhomology-mediated end joining (MMEJ)^1^.

HDR is a faithful repair pathway that mediates DSB repair by utilizing homologous sister chromatids or exogenous homologous donors as templates^2^. CRISPR/Cas9-induced DSBs at pre-selected sites promote HDR-mediated knock-ins (KIs) at nearby regions with plasmid donors harboring long homologous arms (HAs, 500–3000 bp) flanking the foreign DNA^3^. However, the activity of plasmid donor-templated HDR is intrinsically inefficient, which impedes performance of the HDR-based methods in mediating targeted KIs of large transgenes in a variety of cell types, especially in human cells^4,5^. Several studies have reported that single-stranded oligo deoxyribonucleotides (ssODNs) can be used as donor templates to achieve high KI efficiencies, but they can only convey short edits (< 50 bases)^6,7^.

NHEJ is a homology-independent, error-prone repair pathway^8^. Unlike HDR, the NHEJ repair pathway can occur throughout the cell-cycle and is a predominant mechanism to repair DSBs in many types of mammalian cells, including non-dividing somatic cells such as neurons and muscle cells^5,9^. The NHEJ pathway can also be harnessed to drive site-specific transgene KIs by concurrent cleavage of both the genomic DNA and the NHEJ-donor. By the NHEJ pathway, the excised double-stranded transgene fragment rejoins the genomic DNA at the DSB site in random directions and may leave indels at the junctions^10^. Harnessing the NHEJ pathway, homology-independent targeted integration (HITI) has been shown to enhance transgene integration in the intended direction by the rearrangement of the orientation of the CRISPR/Cas9 targeting spacer sequences in the donor vector^11^. However, unpredictable indels at the junction sites impede the application of the NHEJ-based strategies in mediating in-frame open reading frame fusions.

In addition to HDR and NHEJ, MMEJ has also been exploited to mediate targeted integration of large foreign DNAs^12–14^. The MMEJ-based method achieves accurate incorporation of foreign DNAs at the target locus independently of long-range HAs. However, recent reports have demonstrated that the MMEJ-based approach is generally inefficient in mammalian cells^11,15^.

Prime editing is a novel gene editing tool developed to manipulate small deletions, mutations, and insertions^16^. A reverse transcriptase (RT) is conjugated to an spCas9 nickase (H840A) to form PE–Cas9 that nicks the genomic target site and extends a micro-homologous flap (MHF) along with the intended edit at the 3′-end of the cleaved strand. The short edit is then integrated into the target site through flap exchange and mismatch repair independent of DSB. Motivated by the search-and-replace working manner of prime editing and the hypothesis that transgenes modified with single-stranded homologous arms might facilitate targeted KIs, we deduced an application of the prime editor (PE) in donor vector processing and devised a PE-based KI strategy: primed micro-homologues-assisted integration (PAINT).

The PAINT method, initially exemplified by PAINT 1.0, mediated high-efficiency targeted KIs boosted by the reverse-transcribed single-stranded micro-homologous overhangs (MHOs) at each side of the transgene cassette. Optimization of the prime editing guide RNAs (pegRNAs) further enhanced its KI efficacy. Manipulating the mechanisms that control cell-cycle and DNA repair suggested a primed micro-homologues-mediated end joining (PMEJ) pathway that dictates PAINT-mediated targeted KIs in a “copy and paste” manner. This finding inspired us to replace MHOs with MHFs to avoid NHEJ-based imprecise integrations of linearized double-stranded DNAs (ldsDNAs). The finalized version of PAINT, PAINT 3.0, further combined a single MHF and a double-stranded HA to deal with scarless in-frame transgene integrations and achieved high fidelity KIs with minimized on- and off-target integration errors.

Finally, PAINT 3.0 was exploited for targeted KIs in therapeutically relevant genomic loci. PAINT 3.0-mediated non-viral genome targeting at the *TRAC* locus in primary T cells produced as much as 50%–60% functional chimeric antigen receptors (CAR)-T cells, which exhibited specific killing activities when cocultured with tumor cells. We establish that the PAINT strategy is a promising gene editing tool that offers a new opportunity to overcome the barriers that hinder the development of genome engineering technologies.

## Results

### CRISPR-PAINT design and its application in targeted transgene integrations

**Fig. 1:**
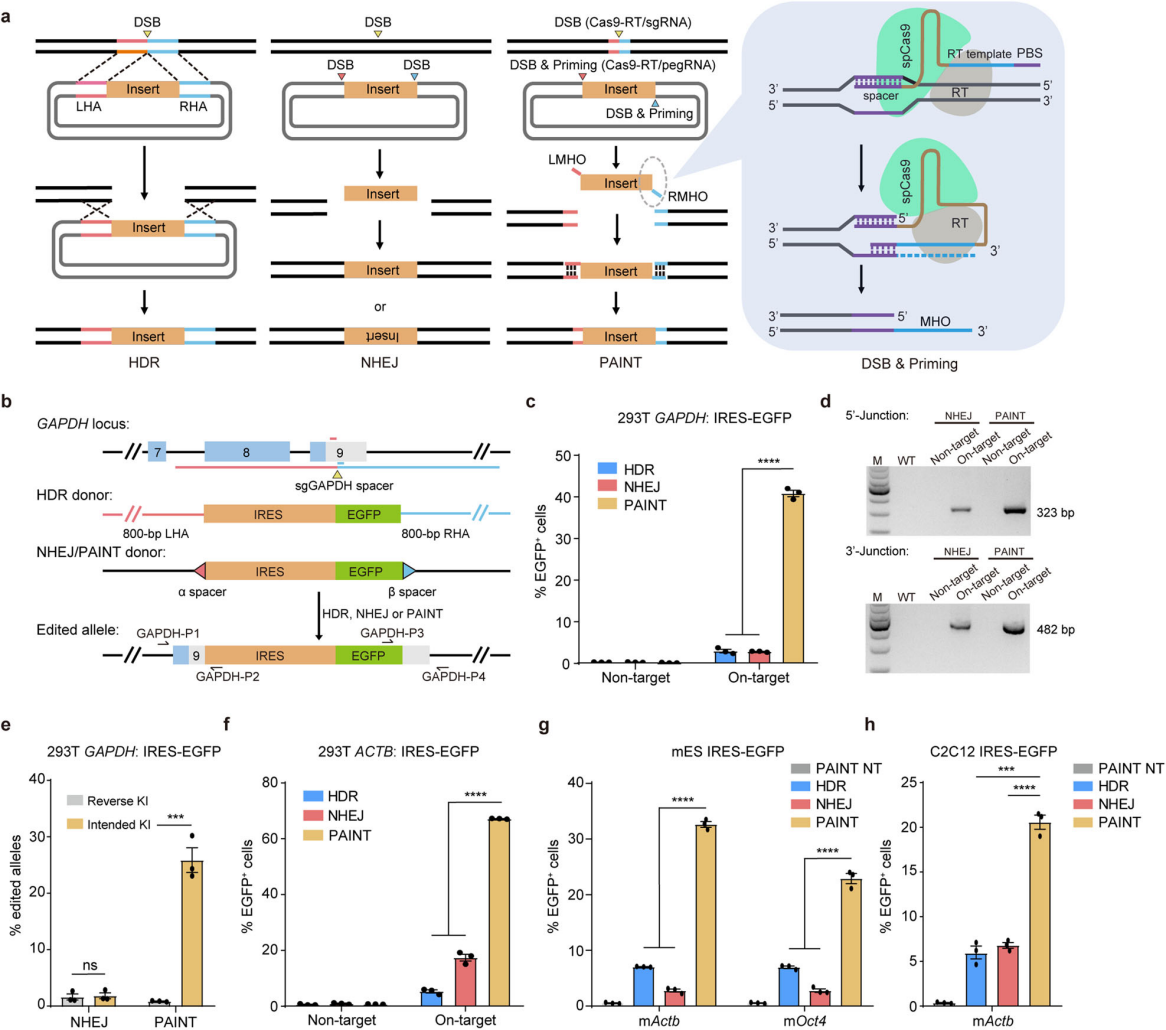
Primed micro-homologous overhangs induce highly efficient targeted transgene integrations. **a** Diagram shows HDR-, NHEJ-, and PAINT-mediated targeted integration of foreign DNAs. Each line corresponds to a DNA strand. LHA and LMH are marked in red; RHA and RMH are marked in cyan. Triangles mark the specific cleavage sites in the genome and donors. Comb teethes represent the annealing of homologous strands. HDR-based integration (first from the left) requires long homologous arms typically ranging from 500–2000 bp, and is facilitated by the introduction of a DSB at the genomic target site. The NHEJ-base method (second from the left) requires concurrent excision of the transgene cassette and cleavage of the genomic DNA at the target site. The linearized double-stranded transgene then integrates into the genomic target site bidirectionally via the NHEJ repair pathway. For the PAINT method (second from the right), both 3′-ends of the excised transgene are modified with single-stranded micro-homologous overhangs (MHOs) by the spCas9–RT/pegRNA system, which then dictate efficient and unidirectional integration of the transgene. The last panel is a diagram showing the synthesis of the right 3′-MHO at the end of the excised transgene. LHA left homologous arm, RHA right homologous arm, LMHO left micro-homologous overhang, RMHO right micro-homologous overhang, RT reverse transcriptase, PBS primer binding site. **b** Diagram shows HDR-, NHEJ-, and PAINT-mediated integration of an IRES-EGFP reporter transgene at the 3′-UTR of human *GAPDH* gene. The yellow triangle marks the genomic target site recognized by sgGAPDH. The long red and cyan lines indicate the 800-bp left and right homologous arms (HAs), respectively. The short red and cyan lines indicate the 35-bp left and right micro-homologues (MHs), respectively. The red and cyan triangles indicate the generic spacers targeted by sgα (or pegα) and sgβ (or pegβ), respectively. PegRNAs are designed corresponding to the MHs and spacers. Correctly edited cells express EGFP and can be observed under a fluorescence microscope or analyzed by FACS. Positions of primers for PCR genotyping are shown by arrows. **c** Editing efficiencies of HDR-, NHEJ-, and PAINT-mediated IRES-EGFP integration at the 3′-UTR of *GAPDH*. **d** Genotyping of NHEJ- and PAINT-edited cells by PCR. Primers GAPDH-P1/GAPDH-P2 and GAPDH-P3/GAPDH-P4 amplify the 5′ junction (323 bp) and the 3′ junction (482 bp) on correctly edited *GAPDH* alleles, respectively. **e** Editing efficiencies of NHEJ- and PAINT-mediated IRES-EGFP integration at the 3′-UTR of *GAPDH* measured by droplet digital PCR (ddPCR). The percentages of edited alleles with intended and reverse IRES-EGFP KIs were analyzed. Three replicates were performed. The results are presented as the mean ± SEM. ns no significance, ****P < 0.0001, unpaired Student’s *t*-test, two-sided. **f** Editing efficiencies of HDR-, NHEJ-, and PAINT-mediated IRES-EGFP integration at the 3′-UTR of human *ACTB* gene in 293T cells. **g** Editing efficiencies of HDR-, NHEJ-, and PAINT-mediated IRES-EGFP integration at the 3′-UTR of *Actb* and *Oct4* genes in mouse ESCs. **h** Editing efficiencies of HDR-, NHEJ-, and PAINT-mediated IRES-EGFP integration at the 3′-UTR of *Actb* in C2C12 cells. For **c**, **f**–**h**, KI efficiencies were presented as the percentage of EGFP^+^ cells among total transfected cells. Three replicates were performed. The results are presented as the mean ± SEM. ns no significance, *P < 0.05, **P < 0.01, ***P < 0.001, ****P < 0.0001, unpaired Student’ s *t*-test, two-sided.

Inserting exogenous DNA fragments free of homologous arms into the genomic target site is inefficient and imprecise^17^. We reasoned that the modification of single-stranded MHOs flanking each end of the transgene can facilitate targeted KIs. To generate the MHO-modified transgene cassette, we first conjugated the murine leukemia virus reverse transcriptase (MLV RT)^16^ to a fully functional spCas9 to form the spCas9–MLV RT fusion protein (hereafter referred to as spCas9–RT, so as to be distinguished from PE–Cas9). Targeting spCas9–RT/sgRNAs to an EGFP coding region in a 293T-EGFP cell line demonstrated that the conjugated RT has no noticeable influence on the catalytic activity of spCas9 (Supplementary Fig. S1a). We then devised the PAINT method by targeting spCas9–RT/pegRNAs to the PAINT-donor that harbors two generic spCas9–RT/pegRNA recognition sequences flanking the transgene cassette. The RT-template of each pegRNA is designed to be homologous to the genomic target site. In PAINT-mediated KI, the transgene is excised from the donor vector and single-stranded MHOs on each side of its 3′-end are generated. With the introduction of a site-specific DSB in the genome using an additional sgRNA, the MHOs-modified transgene is expected to be integrated into the genomic target site efficiently in an orientation-specific manner (Fig. 1a). To evaluate the editing efficacy of the PAINT approach, we integrated a promoterless IRES-EGFP reporter transgene into the 3′-UTR of the human *GAPDH* gene with three approaches including HDR-, NHEJ-based KIs and PAINT (Fig. 1b). Human 293T cells were transfected with the HDR-, NHEJ-, and PAINT-plasmid constructs, and successfully transfected cells were sorted and cultured for 5 more days before fluorescence-activated cell sorting (FACS) analysis. The KI efficiencies were presented as the frequencies of EGFP^+^ cells. The results showed that the IRES-EGFP targeting efficiencies by HDR- and NHEJ-based methods were similarly low (2.99 ± 0.42% and 2.85 ± 0.07%, respectively). Excitingly, the PAINT strategy exhibited outstanding performance showing 40.8 ± 0.76% transfected cells positive for EGFP, a more-than-tenfold increase in editing efficacy over HDR- and NHEJ-mediated approaches (Fig. 1c; Supplementary Fig. S1b). Genotyping of edited cells confirmed PAINT-mediated on-target transgene integration at the 3′-UTR of *GAPDH* (Fig. 1d). Moreover, compared with NHEJ, Sanger sequencing detected more accurate transgene integration junctions at both 5′ and 3′ sides in PAINT-mediated KI (Supplementary Fig. S3a). We also examined the frequencies of unintended on-target integration events (reverse transgene KI and backbone KIs) by digital droplet PCR. The results demonstrated that PAINT method mediated targeted transgene integration in an orientation-specific manner (intended transgene KI = 26.02 ± 2.20%, reverse transgene KI = 1.00 ± 0.06%, backbone KI = 2.10 ± 0.10%, and reverse backbone KI = 5.98 ± 0.19%, respectively). In contrast, NHEJ-based method manifested inefficient intended transgene integration, with a frequency similar to those of undesired KIs (intended transgene KI = 1.97 ± 0.53%, reverse transgene KI = 1.76 ± 0.53%, backbone KI = 2.16 ± 0.34%, and reverse backbone KI = 2.06 ± 0.43%, respectively) (Fig. 1e; Supplementary Fig S1c, d). Taken together, these results showed that the PAINT strategy exhibited robust targeting efficacy, high junction accuracy, and high orientation-specificity in mediating targeted transgene integrations in human cells.

### Enhancing PAINT-mediated targeted KIs by optimized pegRNAs

Several groups have enhanced performance of prime editing by screening and optimizing pegRNA structures and constitutions^16,18–20^. We reasoned that sufficient synthesis and appropriate length of the flanking 3′-MHOs were key to PAINT-mediated targeted KIs. Thus, we sought to further improve the efficiency of PAINT-mediated KIs with optimized parameters of the pegRNAs. We first constructed a series of pegRNAs with varying RT-template lengths ranging from 15 to 45 nt to conduct IRES-EGFP integration at the *GAPDH* locus. FACS analysis showed that pegRNAs with 35-nt RT-template achieved the highest KI frequency (43.53 ± 0.66%), and pegRNAs with 25-nt RT-template showed modestly, but not significantly, lower KI efficiency (39.46 ± 2.11%). However, pegRNAs with 15-nt or 45-nt RT-templates were relatively inefficient (25.33 ± 0.78% and 28.83 ± 0.81%, respectively) (Supplementary Fig. S2a). The results indicated that the optimal length of the RT-template in PAINT-pegRNAs was approximately 35 nt. Of note, the pegRNAs in the PE system usually achieve the highest genome editing efficiency with a much shorter RT-template (approximately 13–19 nt in length)^16^. We also compared PAINT-mediated KI efficiencies with pegRNAs of different primer binding site (PBS) lengths. We found that the PBS sequence as short as 11–12 nt in length was usually sufficient for PAINT-mediated KI, while longer PBS sequences (more than 13 nt in length) might result in declined integration efficiency (Supplementary Fig. S2b–e). These results were in consistent with that of the PE system, in which longer PBS was more prone to cause pegRNA circulation^19^. Hereafter, to avoid extensive screening of pegRNA constructs, we set the RT-template length at 35 nt and the PBS length at 11–12 nt in all subsequent experiments, unless mentioned otherwise. The PAINT-donor harbors a pair of generic spCas9-targeting spacers for transgene excision and MHO modification (Fig. 1a, b). We then introduced two additional generic spacers with high spCas9-targeting activities (Supplementary Fig. S1a) and examined the efficiencies of PAINT-mediated integrations with various spacer pairs. We detected variations in PAINT-mediated targeting efficiencies at *GAPDH* with different spacer pairs (from 34.57 ± 1.40% to 62.67 ± 0.76%) (Supplementary Fig. S2f). We reasoned that the innate activity of the spacer, as well as the influence of the extended region (consists of the RT-template and PBS) on the secondary structure of the pegRNA, resulted in varying MHO-priming efficiencies. We also examined the influence of transgene size on the efficiency of PAINT-mediated KI. We incorporated intron sequences into the EGFP coding region to generate IRES-EGFP reporter transgenes of different sizes (ranging from 1.5 to 6.9 kb; Supplementary Table S2) and performed PAINT-mediated transgene integrations at the *GAPDH* locus. The results showed that the length of the transgene at this range had no substantial influence on the editing efficiency of PAINT-mediated KI (Supplementary Fig. S2h). We then tested PAINT-mediated targeted KIs at other genomic loci across different cell types with optimized pegRNAs. Comparison of PAINT-mediated IRES-EGFP KI with HDR- and NHEJ-based methods at the 3′-UTR of *ACTB* locus (Supplementary Fig. S3b) showed that PAINT enhanced KI frequencies (67.17 ± 0.09%) by 12-fold and 4-fold compared to HDR-based and NHEJ-based strategies, respectively (Fig. 1f; Supplementary Fig. S2g). Genotyping and Sanger sequencing further confirmed PAINT-mediated accurate on-target transgene integrations at both 5′- and 3′-junctions (Supplementary Fig. S3c, d). We then examined PAINT-mediated IRES-EGFP KI efficiencies at the 3′-UTR of *Actb* and *Oct4* in mouse embryonic stem cells (ESCs) and a myoblast cell line C2C12 (Supplementary Fig. S3e, h). Results showed that the PAINT method performed outstandingly in both cell lines compared with NHEJ- and HDR-based methods (Fig. 1g, h; Supplementary Fig. S3f, g, i, j). Together, our results showed that the optimized pegRNAs enabled PAINT-mediated targeted KIs with high editing efficiency and junction accuracy across various genomic loci and cell types.

### Mechanism of PAINT-mediated KI

**Fig. 2:**
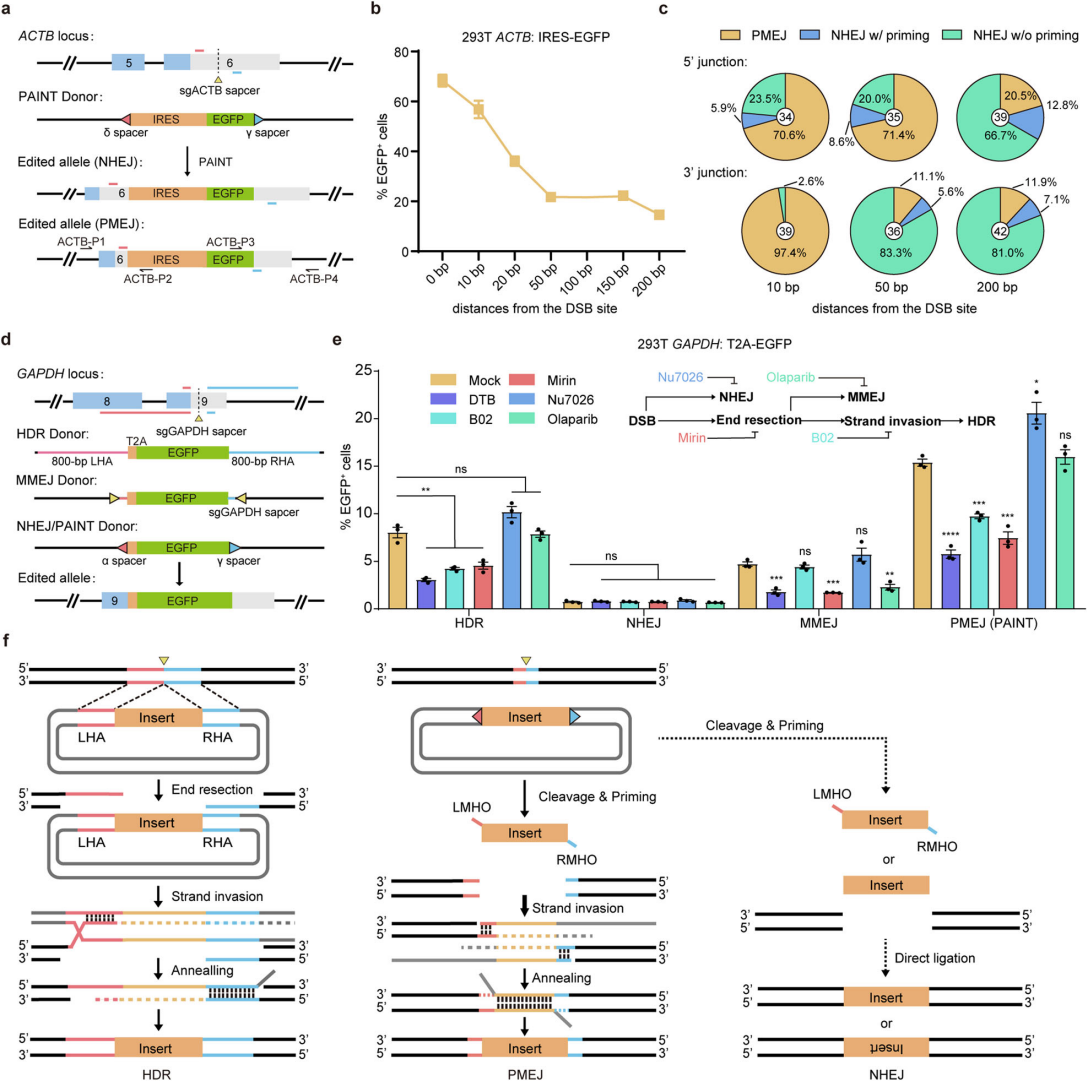
Mechanisms of PAINT-mediated targeted KIs. **a** Diagram shows PAINT-mediated IRES-EGFP integration at the 3′-UTR of human *ACTB* gene with inner MHs. The yellow triangle marks the genomic target site recognized by sgACTB. The short red and cyan lines indicate the 35-bp left and right MHs, respectively. There leaves a distance (ranging from 10 bp to 200 bp) between each MH and the genomic cleavage site. The red and cyan triangles indicate the spacers targeted by pegδ and pegγ, respectively. Theoretically, the transgene may either directly incorporate into the genomic cleavage site by NHEJ, or integrate precisely at the inner MH site by primed PMEJ. Both NHEJ-based incorporation or PMEJ-mediated KI resulted in EGFP expression under the promoter of *ACTB* gene. Positions of primers for PCR genotyping are shown by arrows. **b** Editing efficiencies of PAINT-mediated IRES-EGFP integrations at the 3′-UTR of *ACTB* with inner MHs of various distances away from the cleavage site. The editing efficiency declines as the distance extends. **c** Sanger sequencing analysis of edited junctions confirms the participation of both NHEJ and PMEJ pathways in PAINT-mediated targeted transgene integration. The portion of PMEJ-based integration declines as the distance between the MHs and the genomic cleavage site extends. **d** Diagram shows HDR-, NHEJ-, MMEJ-, and PAINT-mediated T2A-EGFP integration at the 3′-UTR of *GAPDH*. The yellow triangle marks the genomic target site recognized by sgGAPDH. The long red and cyan lines indicate the 800-bp left and right HAs, respectively. The short red and cyan lines indicate the left and right MHs (35 bp for PAINT and 20 bp for MMEJ), respectively. There is a 59-bp distance between the integration target site and the genomic cleavage site. The red and cyan triangles indicate the spacers targeted by sgα (or pegα) and sgγ (or pegγ), respectively. Precisely edited cells express EGFP in-frame of the *GAPDH* coding sequence. **e** Editing efficiencies of HDR-, NHEJ-, MMEJ-, and PAINT-mediated T2A-EGFP integration at the 3′-UTR of *GAPDH* treated with chemical inhibitors of the cell-cycle or DNA repair pathways. **f** Diagram compares the working models of plasmid donor-templated HDR and PAINT. Each line corresponds to a DNA strand. LHA and LMH are marked in red; RHA and RMH are marked in cyan. Triangles mark the specific cleavage sites in the genome and donors. Comb teethes represent the annealing of homologous strands. The left panel shows the SDSA model of plasmid donor-templated HDR. There are three central steps of the SDSA pathway. End resection, the 3′-ended DNA strand is resected at the break to create a 3′ single-stranded HA. Strand invasion, the single-stranded HA invades the plasmid donor by displacing one strand of the homologous DNA duplex and pairing with the other. This step then initiates donor-templated DNA repair synthesis. Annealing, the synthesized HA homologue then repairs with the homologous genomic DNA end to mediate DNA end rejoining. For plasmid donor-templated HDR, extended DNA end resection is a crucial step but is rate-limiting. The right panel presents a working model of PAINT method. PAINT achieves high-efficiency targeted transgene integrations mainly through PMEJ, an SDSA mechanism similar to HDR. In this PMEJ model, it is the single-stranded MHOs of the primed donor that invades the homologous genomic DNA ends to initiate DNA repair synthesis. This process is independent of extended genomic DNA end resection and, thus, may contribute to elevated KI efficiencies. A small portion of NHEJ-based transgene incorporation may also be involved in PAINT-mediated targeted KI. LHA left homologous arm, RHA right homologous arm, LMHO left micro-homologous overhang, RMHO right micro-homologous overhang. For **b**, **e**, editing efficiencies were measured as the percentage of EGFP^+^ cells among total transfected cells. Three replicates were performed. The results are presented as the mean ± SEM. ns no significance, *P < 0.05, **P < 0.01, ***P < 0.001, ****P < 0.0001, unpaired Student’ s *t*-test, two-sided.

We first explored whether the PAINT method can mediate targeted KI with inner MHs that left a distance between the intended edit site and the cut site. We performed PAINT-mediated IRES-EGFP transgene integration at the 3′-UTR of *ACTB* with pegRNAs targeting inner MHs (10 bp to 200 bp away from the genomic cleavage site) and evaluated the editing efficiencies by measurement of EGFP^+^ cells (Fig. 2a). Results showed that the KI frequencies dropped drastically as the distance between the MH site and the cleavage site increased (from 68.33 ± 1.36% at 0 bp to 14.7 ± 0.60% at 200 bp) (Fig. 2b). Sanger sequencing analysis revealed the constitution of two integration events: NHEJ-based direct incorporation at the genomic cleavage site and PMEJ at the MH site. Decreased KI frequency was also coupled with reduction in PMEJ-mediated edits and increase in NHEJ-mediated edits along with the extension of the spacer between the MH site and the cut site (Fig. 2c; Supplementary Fig. S4a, b). These results demonstrated that PMEJ dominated PAINT-mediated KI with outer MHs (< 10 bp), and contributed to the high editing efficiency of PAINT method. PMEJ might integrate the primed transgene into the target site in two ways: a “cut and paste” manner, that is, the primed transgene is directly incorporated into the target site without transgene-templated DNA synthesis; a “copy and paste” manner, that is, the primed transgene is used as a template for DNA synthesis-dependent DSB repair. Examining the mechanism of PMEJ with chemical inhibitors of the cell-cycle and DNA repair pathways, we first ruled out the contribution of NHEJ-mediated KI in PAINT by targeting a T2A-EGFP reporter transgene into the *GAPDH* locus (Fig. 2d). As there is a distance between the cut site and the coding region of *GAPDH*, we reasoned that only PMEJ-mediated precise in-frame KI could result in expression of the EGFP protein and be detected by FACS analysis, while NHEJ-based transgene incorporation resulted in non-in-frame targeting and was negative for EGFP expression. As expected, the results showed that the NHEJ-based method achieved EGFP^+^ frequencies at the background level, whether treated with inhibitors or not. EGFP^+^ frequencies of PAINT-mediated transgene integrations were reduced along with the blockage of the cell-cycle at the G1–S boundary using thymidine (double thymidine blockage, DTB), or inhibition of the HDR pathway regulators such as RAD51 (with the inhibitor B02) and the Mre11–Rad50–Nbs1complex (with the inhibitor mirin). Inhibition of DNA-PK with Nu7026 resulted in slightly enhanced PAINT-mediated in-frame KI, probably due to the restrained competing NHEJ pathway. However, the MMEJ pathway inhibitor, Olaparib, had no substantial influence on the editing efficiencies of PAINT and the HDR-based method (Fig. 2e). As RAD51 was reported to mediate the search for a template during HDR^21,22^, we therefore speculated that although the MHO-modified PAINT-donor structurally resembled the MMEJ-donor, the PMEJ pathway was more likely to work in a “copy and paste” model similar to HDR, rather than a “cut and paste” model of MMEJ or NHEJ. To investigate why PAINT is much more efficient than plasmid donor-templated HDR in mediating targeted transgene integrations, we used a plasmid donor, a double-armed ssODN donor and a sticky-ended short dsDNA donor (a mimic of the primed donor) to recover a mutant EGFP-expressing gene in a 293T-ΔEGFP cell line. The 293T-ΔEGFP cell harbors an EGFP coding gene with a 13-bp deletion that causes a frameshift and premature termination. Correctly edited cells that precisely integrate the 13-bp edit into the mutant locus can express EGFP and be identified by FACS (Supplementary Fig. S5a). For plasmid donor-templated HDR, long-range end resection is essential for the strand invasion step but is rate-limiting. ssODNs have been reported to work in a synthesis dependent strand annealing (SDSA) model independent of extensive genomic DNA end resection, thus achieving higher editing efficiencies than plasmid donors^23^. Consistently, our results showed that both double-armed ssODN and sticky-ended dsDNA achieved high gene recovery efficiencies, while plasmid donor-templated HDR resulted in relatively low editing efficiency (Supplementary Fig. S5b, c). Sanger sequencing further confirmed precise editing with the sticky-ended dsDNA (Supplementary Fig. S5d). We thus speculated that the sticky-ended short dsDNA and PAINT-primed donor work in similar ways and proposed the long-range end resection-independent SDSA mechanism of PMEJ (Fig. 2f; Supplementary Fig. S5e).

### PAINT with micro-homologous flaps

**Fig. 3:**
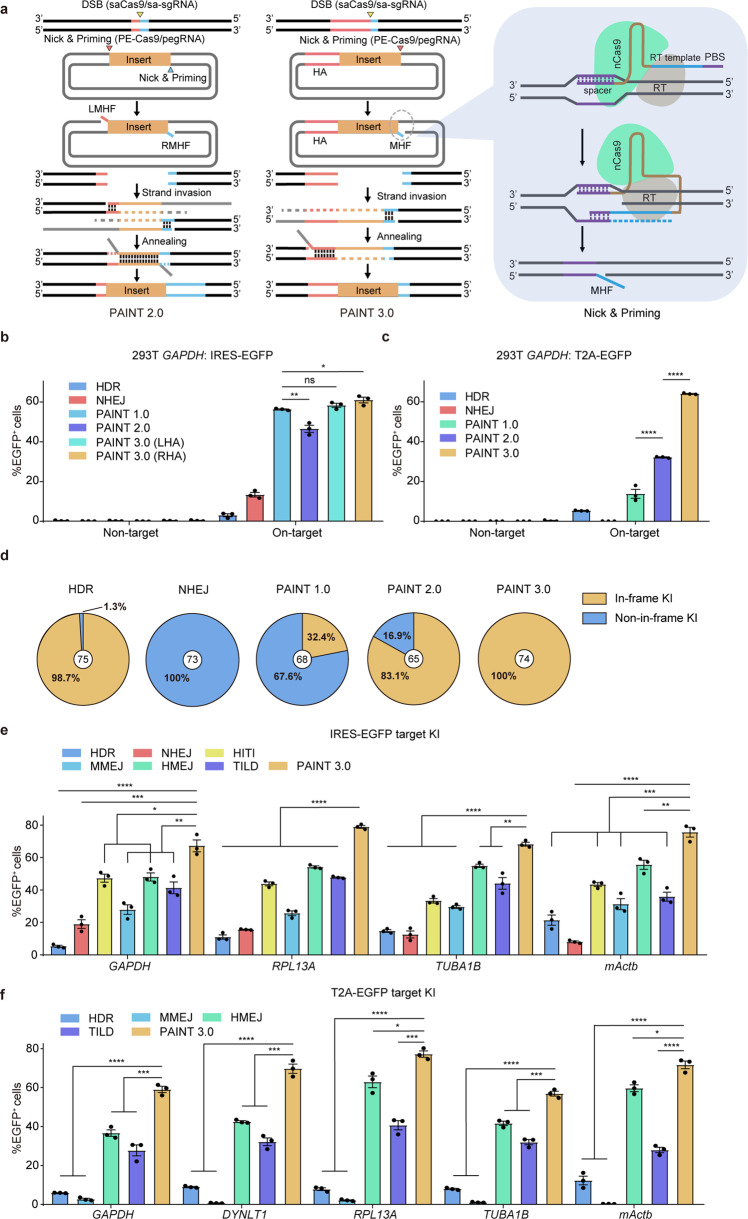
Targeted transgene integration mediated by primed micro-homologous flaps. **a** Diagram shows strategies of the PAINT 2.0 and PAINT 3.0 methods. Each line corresponds to a DNA strand. LHA and LMH are marked in red; RHA and RMH are marked in cyan. Triangles mark the specific cleavage sites in the genome and donors. Comb teethes represent the annealing of homologous strands. Both PAINT 2.0 and PAINT 3.0 utilize the PE2 (PE–Cas9/pegRNA) system for donor processing to generate MHFs. An additional programable nuclease, e.g., saCas9 is applied to introduce DSBs at the genomic target site. The left panel shows PAINT 2.0-mediated targeted KIs. A pair of 3′-MHFs flanking the transgene participates in targeted transgene integration without donor linearization, thus avoiding NHEJ-mediated imprecise KIs. The middle panel presents PAINT 3.0-mediated targeted KIs. The transgene cassette is sandwiched by a 3′-MHF on one side and a double-stranded HA on the other side. The 3′-MHF triggers strand invasion of the transgene while the HA mediates strand annealing to ligate the two genomic DNA parts. The right panel shows the generation of the 3′-MHF via a nicking & priming process. LMHF left micro-homologous flap, RMHF right micro-homologous flap. **b** Editing efficiencies of HDR-, NHEJ-, PAINT 1.0-, PAINT 2.0-, PAINT 3.0 (LHA)-, and PAINT 3.0 (RHA)-mediated IRES-EGFP integration at the 3′-UTR of *GAPDH*. **c** Editing efficiencies of HDR-, NHEJ-, PAINT 1.0-, PAINT 2.0-, and PAINT 3.0-mediated T2A-EGFP integration at the 3′-UTR of *GAPDH*. **d** Analysis of 3′-junctions of edited alleles by Sanger sequencing shows portions of in-frame and non-in-frame KIs with different methods. **e** Side by side comparison of the editing efficiencies of HDR-, NHEJ-, HITI-, MMEJ-, HMEJ-, TILD-, and PAINT 3.0-mediated IRES-EGFP integration at the 3′-UTR of various housekeeping genes. **f** Side-by-side comparison of the editing efficiencies of HDR-, MMEJ-, HMEJ-, TILD-, and PAINT 3.0-mediated precise in-frame T2A-EGFP integration at the 3′-UTR of various housekeeping genes. For **b**, **c**, **e**, **f**, editing efficiencies were measured as the percentage of EGFP^+^ cells among total transfected cells. Three replicates were performed. The results are presented as the mean ± SEM. ns no significance, *P < 0. 05, **P < 0. 01, ***P < 0.001, ****P < 0.0001, unpaired Student’ s *t*-test, two-sided.

The initially designed PAINT strategy, hereafter referred to as PAINT 1.0, exploits the spCas9–RT/pegRNA system in donor processing and generates linearized double-stranded transgene cassette modified with single-stranded 3′-MHOs to promote efficient targeted transgene integrations. However, the present of ldsDNAs in PAINT-edited cells may lead to undesired on-target KIs such as reverse KI, backbone KI and non-in-frame KI mediated by NHEJ (Figs. 1e, 2c; Supplementary Fig. S1d). Moreover, ldsDNAs may also incorporate randomly into artificially triggered or endogenous off-target DSB sites, leading to the risk of tumorigenesis. To solve these problems, we utilized PE2 (PE–Cas9/pegRNA) to introduce nicks and MHFs to the PAINT-donor and developed PAINT 2.0. We designed PE–Cas9-coupled pegRNAs following the optimized parameters determined in PAINT 1.0. The PAINT 3.0 method further replaced one of the MHFs with a long-range (800 bp) double-stranded HA to deal with scarless in-frame integrations. The HA sequence of the PAINT 3.0-donor locates either upstream (left homologous arm, LHA) or downstream of the transgene (right homologous arm, RHA) depending on the desired editing outcome. Both PAINT 2.0 and PAINT 3.0 methods prevent the generation of ldsDNAs and are expected to achieve minimized on-target undesired KIs and ectopic random integrations. An extra programable nuclease, for example, saCas9 was exploited to introduce DSBs at the genomic target loci (Fig. 3a). To evaluate the editing efficiencies of the PAINT 2.0 and PAINT 3.0 methods, we first targeted the IRES-EGFP transgene into the 3′-UTR of human *GAPDH* gene by the PAINT approaches and traditional NHEJ- and HDR-based methods (Supplementary Fig. S6a). We detected a lightly reduced editing efficiency of PAINT 2.0 compared with that of PAINT 1.0, which might due to the absence of NHEJ-contributed KI in the PAINT 2.0 method. However, PAINT 3.0 rescued the editing efficiency via the MHF/HA combination (Fig. 3b; Supplementary Fig. S6b). PCR genotyping and Sanger sequencing confirmed on-target integrations with accurate junctions mediated by PAINT 2.0 and PAINT 3.0 (Supplementary Fig. S6c–e). We then examined the editing efficiencies of the PAINT methods in precise in-frame KI by targeting a T2A-EGFP reporter transgene into the same *GAPDH* locus (Supplementary Fig. S7a). FACS analysis showed that PAINT 3.0 achieved the highest editing efficiency, demonstrating the superiority of the MHF/HA combination compared with double MHFs (PAINT 2.0) or MHOs (PAINT 1.0) in mediating precise in-frame transgene integrations (Fig. 3c). Moreover, Sanger sequencing analysis of the KI junctions revealed that PAINT 3.0 achieved minimized non-in-frame transgene integrations (Fig. 3d; Supplementary Fig. S7b–f). In addition, compared with the HDR method, we also detected increased KI/indels ratio in PAINT 3.0-mediated T2A-EGFP integration at the *GAPDH* locus (Supplementary Fig. S8). To evaluate the off-target editing effects of the PAINT systems, we targeted the IRES-EGFP transgene to an artificial off-target site (with editors designed to target the *GAPDH* locus except an sgRNA targeting the 3′-UTR of *RPL13A*), and detected the frequencies of EGFP^+^ cells (Supplementary Fig. S9a). The results showed that the PAINT 2.0 and PAINT 3.0 methods achieved minimized off-target KI frequencies compared with NHEJ and PAINT 1.0 (Supplementary Fig. S9b, c). To further evaluate the editing efficacy of the PAINT 3.0 method on a larger scale, we performed side-by-side comparisons with different KI strategies (Supplementary Fig. S9d) by targeting the IRES-EGFP and T2A-EGFP transgenes into several housekeeping genes including *GAPDH*, *PRL13A*, *TUBA1B*, *DYNLT1*, and mouse *Actb*. Our results showed that the PAINT 3.0 method achieved significantly highest editing efficiencies across all target sites and cell types tested (Fig. 3e, f). Consistent with PAINT 1.0, PAINT 3.0 achieved the highest KI efficiencies with pegRNAs of 35-nt RT template length at various genomic loci (Supplementary Fig. S10a, b). To detect the generation of single-stranded MHs on primed donors, we developed a PCR-based method to amplify the MHF region with specific primers in cells co-transfected with the PE editor and the PAINT-donor (Supplementary Fig. S10c). Specific PCR bands were detected on PE-primed donors with pegRNAs targeting *GAPDH*, *RPL13A*, and *TUBA1B* (Supplementary Fig. S10d). Sanger sequencing of the PCR products also confirmed pegRNA-templated elongation of single-stranded MHs on the nicked DNA strands of PAINT-donors (Supplementary Fig. S10e). Together, our results established that the finalized version of PAINT, PAINT 3.0, mediated targeted transgene integrations with high-efficiency, high-accuracy, and high-precision in mammalian cells.

### PAINT 3.0 editing at therapeutically relevant genomic loci

**Fig. 4:**
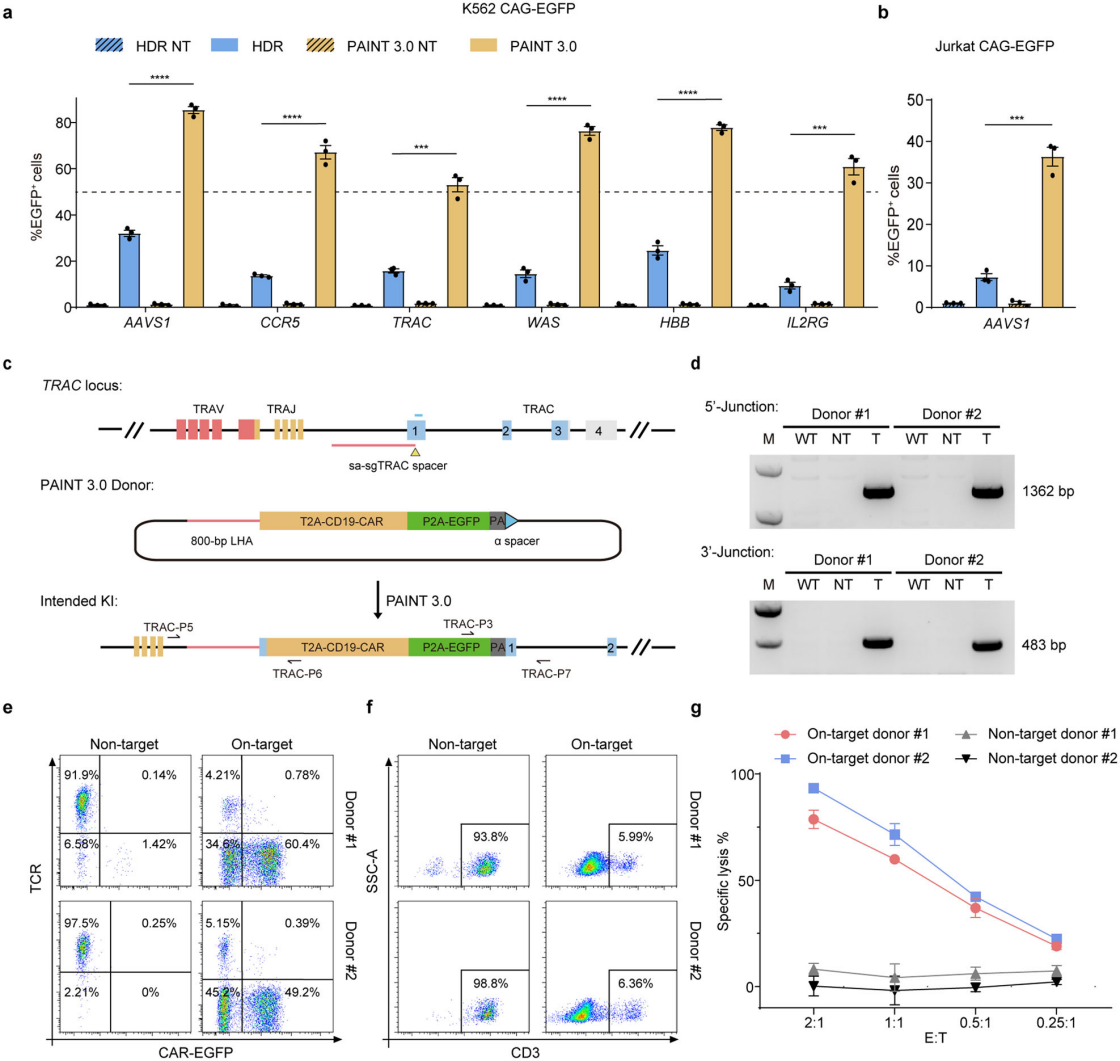
PAINT 3.0 mediates high-efficiency targeted KIs at therapeutically relevant genomic loci. **a** Editing efficiencies of PAINT 3.0-mediated CAG-EGFP integration at safe harbors and inherited disease-associated genes in K562 cells. **b** Editing efficiencies of PAINT 3.0-mediated CAG-EGFP integration at the *AAVS1* locus in Jurkat cells. **c** Diagram shows PAINT 3.0-mediated targeted integration of a CD19 CAR-EGFP transgene cassette at the *TRAC* locus in primary T cells. Yellow triangle marks the genomic target site recognized by sa-sgTRAC. Red and cyan lines indicate the 800-bp left HA and the 35-bp right MH, respectively. Cyan triangle indicates the spacer targeted by pegα. Precisely edited cells express CD19 CAR and EGFP under the regulation of the TCR promoter. Positions of primers for genotyping are shown by arrows. **d** PCR genotyping of PAINT 3.0-edited CAR-T cells. Primers TRAC-P5/ TRAC-P6 and TRAC-P3/TRAC-P7 amplify the 3′ junction (1362 bp) and the 3′ junction (483 bp) of correctly edited *TRAC* alleles, respectively. **e** FACS scatter plots show high-efficiency PAINT 3.0-mediated editing in primary T cells. **f** FACS scatter plots show CD3 expression in edited CAR-T cells. **g** Killing ability of PAINT 3.0-edited CAR-T cells measured by coculture with target tumor cells. For **a**, **b**, editing efficiencies were measured as the percentage of EGFP^+^ cells among total transfected cells. Three replicates were performed. The results are presented as the mean ± SEM. ***P < 0.001, ****P < 0.0001, unpaired Student’ s *t*-test, two-sided.

To evaluate the potential of the PAINT method in therapeutic applications, we examined the editing efficiency of PAINT 3.0 at several therapeutically relevant genomic loci. We first targeted a 2.5-kb CAG-EGFP transgene into three identified safe harbor loci including *CCR5*, *AAVS1,* and *TRAC*, and three inherited disease-related genes including *WAS* (associated with Wiskott-Aldrich Syndrome and Thrombocytopenia 1)^24^, *HBB* (associated with Beta-Thalassemia and Sickle Cell Anemia)^25,26^ and *IL2RG* (associated with X-linked severe combined immunodeficiency)^27^ in K562 cells, a myeloid tumor cell line. Impressively, results showed that the PAINT 3.0 method achieved up to 85% editing frequency at the *AAVS1* locus and > 50% editing efficiencies at all genomic loci tested. The genomic target sites in *WAS*, *HBB*, and *IL2RG* genes were near the ATG translation start site, and allowed for an all-in-one coding sequence incorporation treatment to cope with large numbers of heterogenous diseased-related mutations (Fig. 4a; Supplementary Figs. S11, S12). Moreover, PAINT 3.0 also managed a > 30% editing efficiency at the *AAVS1* locus in Jurkat cells, a T cell-derived tumor cell line (Fig. 4b). Finally, we exploited the PAINT system for non-viral genome targeting in primary T cells. PAINT 3.0-mediated in-frame integration of a CD19 CAR-EGFP cassette at the *TRAC* locus generated TCR^–^ CAR-T cells with remarkable efficiencies (60.4% for donor #1 and 49.2% for donor #2), higher than, or at least similar to that of AAV-mediated editing, which faces a manufacturing challenge^28^ and remains a integration-related safety concern^29,30^ (Fig. 4c–f; Supplementary Fig. S13). The edited CAR-T cells expressed CD19 CAR under the regulation of the *TCR* promoter, and exhibited specific killing ability when cocultured with target tumor cells (Fig. 4g). Taken together, these findings showed that the PAINT 3.0 method manifested outstanding performance in genome editing for therapeutically relevant cell types and loci.

## Discussion

Programmable nucleases, especially the rapidly developing CRISPR/Cas9 system, have drastically accelerated the application of genome engineering technologies in many areas, including basic biological research^31,32^, agricultural production^33,34^, and biomedical development^35–37^. Versatile editing tools have been devised to generate site-specific genomic modifications, such as DNA fragment deletion, insertion, replacement, or gene knockout. However, there remains room for the development of gene editing tools to conduct more efficient and precise targeted KIs. Here, we report a newly devised KI strategy, PAINT, which performs outstandingly in mediating targeted integrations of large transgenes in a variety of mammalian cells. We report that the finalized version of PAINT, the PAINT 3.0 method, outperforms existing KI strategies mainly in two aspects. First, PAINT 3.0 exhibits the most robust editing efficacy among all KI strategies tested at various genomic loci, especially in human cells. Second, PAINT 3.0 mediates highly precise KIs with minimized on- and off-target integration errors. The robust KI efficacy and high fidelity of the PAINT approach will shed light on cell and gene therapies for inherited diseases.

Efficiencies and endogenous repair pathway choices in DSB-triggered targeted transgene integrations are largely dependent on the configuration of the provided donor vector. For example, transgenes free of HAs are incorporated into the specific genomic cleavage site mainly via the error-prone NHEJ pathway, while HA-flanked transgenes prefer more precise targeted KIs via homology dependent pathways such as HDR, HMEJ or MMEJ. In our study, we targeted prime editors to donor vectors, rather than the genomic DNA, and generated a distinctive donor configuration: the primed single-stranded micro-homologues flanking the transgene cassette. Our results demonstrated that the 3′-MHOs mediated highly efficient and orientation-specific KIs. In addition, more efficient priming and appropriate length of the MHOs achieved by optimization of the prime editors, especially parameters of the pegRNAs, further enhanced the editing efficacy of the PAINT systems. Treatment with chemical inhibitors of regulators of the cell-cycle and DNA repair pathways revealed a “copy and paste” working model, referred to as PMEJ, in the PAINT strategy. We thus speculate that the MHO structure facilitates efficient targeted KIs by promoting strand invasion independent of the rate-limiting long-range DNA end resection process. PAINT 3.0 combines an MHF and an HA to achieve more efficient and precise targeted KIs, especially in the cases of scarless in-frame transgene integrations. In the PAINT 3.0 method, the MHF mediates the strand invasion process to initiate transgene templated DNA synthesis, while the synthesized homologue of the HA is for strand annealing with the other genomic homologous end. Therefore, a single MHF in PAINT 3.0 is sufficient to support the strand invasion-strand annealing process. In addition, compared with the initial version, PAINT 3.0 introduces a single nick rather than DSBs to avoid linearization of the donor vector, thus increased the safety of the edited cells, especially in clinical applications. Indeed, our results demonstrated that the PAINT 3.0 method exhibited higher editing precision than the initial version.

The major motivation of introducing PE2 (to replace spCas9–RT/pegRNA) in donor processing is to avoid the generation of ldsDNAs, thus to minimize NHEJ-induced on- and off-target integration errors. Both the PAINT 2.0 and PAINT 3.0 methods exploit PE2 (PE–Cas9/pegRNA) for donor processing and require an extra nuclease to generate DSBs at the genomic target loci. Although the introduction of a second nuclease indeed increases the complexity of the PAINT system, the updated versions of PAINT remarkably improve editing efficiency and precision.

Our PAINT method is a creative application of PE in dealing with large transgene KIs. In recent studies, PE coupled with recombinases has been exploited to mediate large transgene integrations^38,39^. However, these methods exhibited limited editing efficiencies. Moreover, the requirement of a landing pad makes them not compatible with precise in-frame KIs.

Overall, our study established that the PAINT system is an effective editing tool that mediates robust integration of large foreign DNAs at various genomic loci in a variety of mammalian cells. We believe that along with the development of more efficient delivery systems for gene editors, such as LNP^40^ and RNP^41^, complexity of the editor components will not be a problem, and that the PAINT 3.0 method, with its high editing efficiency, fidelity, and accuracy, has a great potential to pave a new avenue for the development of genome engineering technologies.

## Materials and methods

### Plasmid construction

To generate the pCAG-spCas9-RT-T2A-mCherry plasmid, a pCAG-spCas9-T2A-mCherry plasmid was digested by AscI and KpnI and gel purified. DNA fragments with 20 bp homologous arms: an N-terminal spCas9 fragment, a T2A-mCherry fragment amplified from the pCAG-Cas9-mCherry plasmid, and an MLV RT fragment amplified from the pCMV-PE2 (#132775, addgene) were cloned into the digested pCAG-spCas9-T2A-mCherry plasmid by HiFi DNA Assembly Master Mix (NEB). To generate the pCAG-PE-Cas9-T2A-mCherry plasmid, the PCR amplicons above were cloned into an AscI/KpnI digested pCAG-spCas9 (H840A)-T2A-mCherry plasmid with HiFi DNA Assembly Master Mix (NEB). All sgRNA plasmids were generated by integrating the annealed spacer oligos into BsaI digested backbones (pUC19-U6-sp-sgRNA Scaffold and pUC19-U6-sa-sgRNA Scaffold) with T4 ligase (NEB). PegRNA plasmids were generated by cloning the extended fragment (containing an RT-template and a PBS sequence) into corresponding HindIII digested sgRNA plasmids using HiFi DNA Assembly Master Mix (NEB). To construct HDR donors, the transgene sandwiched by the left and right HAs amplified from human or mouse genome was cloned into the pGH plasmid linearized by EcoRV. To construct NHEJ/PAINT 1.0/PAINT 2.0 donors, the transgene sandwiched by a pair of 23-nt generic spCas9 target sequences was cloned into the pGH plasmid linearized by EcoRV. To construct PAINT 3.0 donors, the transgene sandwiched by a 23-nt generic spCas9 target sequence and an HA was cloned into the pGH plasmid linearized by EcoRV. To construct scNHEJ and HITI donors, the transgene coupled with a single 23-nt generic or gene-specific spCas9 target sequence was cloned into the pGH plasmid linearized by EcoRV. To construct HMEJ and MMEJ donors, the transgene sandwiched by the left and right HAs (≥ 50 bp) or MHs (20 bp) each coupled with a gene-specific spCas9 target sequence was cloned into the pGH plasmid linearized by EcoRV. TILD donors were amplified by PCR from HDR donors and purified with Monarch PCR & DNA Cleanup Kit (NEB). Synonymous mutations were introduced into the EGFP coding sequence in KI-donors to avoid off-targeting by the generic spacers. Sequences of sgRNA spacers used in this study are listed in Supplementary Table S1. Sequences of transgenes are listed in Supplementary Table S2.

### Cell culture

293T cells (ATCC), C2C12 (ATCC) myoblast cells, K562 cells (ATCC) and Jurkat cells (ATCC) were cultured in 37 °C, 5% CO_2_ in DMEM Medium (Gibco) plus 10% FBS (Gibco), 2 mM GlutaMAX-I (Gibco), and 0.01 mM beta-mercaptothanol (Gibco). Cells were passaged every 2 d. The mouse ESC cell line was established in our own laboratory and was cultured at 37 °C, 5% CO_2_ in N2B27 medium (Gibco) supplemented with GlutaMAX-I (2 mM, Gibco), beta-mercaptothanol (0.01 mM, Gibco), leukocyte inhibition factor (20 ng/mL, R&D systems), CHIR99021 (3 μM, R&D systems), and PD0325901 (1 μM, R&D systems). Mouse ESCs were passaged every 2 d and plated on dishes precoated with Fibronectin (Millipore). Primary T cells were isolated from peripheral blood using the EasySep Human T Cell Isolation Kit (STEMCELL) according to the manufacturer’s instructions. Bulk T cells were cultured in X-Vivo15 medium (STEMCELL) with 5% FBS, 50 μM 2-mercaptoethanol, and 10 μM N-acetyl l-cystine. Immediately after isolation, T cells were stimulated for 3 d with anti-human CD3/CD28 magnetic dynabeads (ThermoFisher) at a beads to cells ratio of 1:1, along with a cytokine cocktail of IL-2 at 200 U/mL (PeproTech), IL-7 at 5 ng/mL (PeproTech), and IL-15 at 5 ng/mL (PeproTech) before electroporation. Electroporated T cells were maintained in culture media with IL-2 at 500 U/mL, and the media was changed every 2 d until the T cells were analyzed for targeted CAR integration efficiency or in vitro killing ability. All cell lines and primary cells were tested negative for mycoplasma contamination.

### Cell transfection

Unless otherwise noted, 293T cells and mouse ESCs were transfected using Lipofectamine 3000 (Invitrogen) following the manufacturer’s instructions. 1 μg spCas9/spCas9-RT/PE-Cas9 plasmid, 0.8 μg saCas9/Cas12a Ultra plasmid, 1 μg donor plasmid, 0.3 μg sgRNA/crRNA plasmid and 0.3 μg pegRNA plasmid were transfected for each well of a 6-well plate according to the method used. For repair pathway and cell-cycle inhibition assay, 1 × 10^6^ 293T cells pre-treated with inhibitors were transfected by electroporation (1400 V, 10 ms, 3 pulses, Invitrogen Neon) with 5 μg spCas9/spCas9-RT plasmid, 5 μg donor plasmid, 1 μg sgRNA plasmid and 1 μg pegRNA plasmid according to the method used. For C2C12 cells, 1 × 10^6^ cells were transfected by electroporation (1600 V, 10 ms, 3 pulses; Invitrogen Neon) with 5 μg spCas9/spCas9-RT plasmid, 5 μg donor plasmid, 1 μg sgRNA plasmid and 1 μg pegRNA plasmid according to the method used. For K562 and Jurkat cells, 1 × 10^6^ cells were transfected by electroporation (SE Cell Line 4D-Nucleofector X Kit, program FF-120 for K562 cells and program CL-120 for Jurkat cells; Lonza) with 5 μg spCas9/PE-Cas9 plasmid, 3 μg saCas9 plasmid, 5 μg donor plasmid, 1 μg sgRNA plasmid and 1 μg pegRNA plasmid according to the method used. For primary T cells, 5 × 10^6^ cells were transfected by electroporation (P3 primary 4D-Nucleofector X Kit, program EO-115; Lonza) with 5 μg PE-Cas9 plasmid, 3 μg saCas9 plasmid, 5 μg donor plasmid, 1 μg sgRNA plasmid and 1 μg pegRNA for PAINT 3.0-mediated targeted integration of a CD19 CAR-EGFP transgene cassette at *TRAC* locus.

### FACS

For adherent cells such as 293T, C2C12, and mouse ESCs, cells were digested with 0.25% trypsin and resuspended with DMEM plus 10% FBS, washed twice with PBS, and resuspended with 500 μL PBS before flow cytometry cell sorting or analysis. For suspension cells such as K562 cells and Jurkat cells, cells were centrifuged, washed, and resuspended with 500 μL PBS before flow cytometry cell sorting or analysis. Before flow cytometry cell analysis, transfected primary T cells were immunostained with antibodies: Brilliant Violet 421 anti-human CD3 Antibody (300434, Biolegend), APC anti-human TCR α/β Antibody (306718, Biolegend) according to the manufacturer’s instructions.

For pre-sorting and collection of successfully transfected cells, sorted mCherry-positive cells were reloaded and were analyzed in the same gating conditions, showing > 98% mCherry-positive. For cell gating, a forward and side scatter plot (FSC × SSC) was used to exclude debris and gate on the cell populations. Cell doublets were gated out on an SSC height and SSC area plot. Subsequently, fluorescence-positive cells were gated on a positivity threshold established using untreated cells. Samples were sorted on a BD FACSAria Fusion system and analyzed by FlowJo (version 7.6).

### Genotyping

Genomic DNA of cell samples was extracted using the Mouse Direct PCR Kit (Bimake) according to the manufacturer’s instructions. Samples were mixed with 100 μL Buffer L and 2 μL Protease Plus, and incubated at 55 °C for 30 min, then 100 °C for 5 min for DNA isolation. Site specific KIs and KI junctions were amplified using specific primers that bound outside of the HAs or MHs. The sizes of the PCR products were identified by agarose gel electrophoresis. Sequences of primers for genotyping are listed in Supplementary Table S3.

### Sanger sequencing analysis

PCR products were purified using a Monarch PCR & DNA cleanup kit (NEB), and then cloned into a pMD18-T vector (TaKaRa) according to the manufacturer’s instructions. Colonies were then picked for plasmid extraction and Sanger sequencing. The Sanger traces were analyzed using the DNAMAN software (Version 7).

### Treatment with inhibitors of cell-cycle and DNA repair pathways

For different repair pathway inhibition assays, cells were pre-treated with B02 (HY-101462, 20 μM; MCE), mirin (HY-117693, 25 μM; MCE), Nu7026 (HY-15719, 2 μM; MCE), or Olaparib (HY-10162, 10 nM; MCE) for 12 h. For the DTB cell-cycle arrest experiment, cells were pre-treated with thymidine (T9250-1G, 2 mM; Sigma) for 18 h, thymidine was removed, the cells were cultured in normal media without thymidine for 9 h and then, thymidine was re-added to the cells for a second round of 18 h. Inhibitor treated cells were transfected using the Neon Transfection System (Invitrogen) as described above. Transfected cells were treated with the inhibitors for another 2 d before cultured in normal media.

### Luciferase-based T cell in vitro killing assay

K562-CD19-luciferase cell-based cytotoxicity was assessed as previously described^42^. Briefly, K562-CD19-luciferase cells were suspended at a density of 1 × 10^5^ cells/mL in RPMI1640 medium and seeded in white opaque plate. Then effector cells were added at the indicated ratio. After incubating at 37 °C in 5% CO_2_ for 24 h, 10 μL Steady-Glo luciferase substrate (Promega) was added, and 5 min later, luminescence was recorded by PerkinElmer Ensight. The results were reported as percentage of killing based on the luciferase activity compared with tumor cells alone (percentage killing = 100 – [(RLU from well with effector and target cell coculture)/(RLU from well with target cells) ×100]).

### Droplet digital PCR

Genomic DNA of edited 293T cells was extracted with the MicroElute Genomic DNA Kit (Omega) according to the manufacturer’ s instructions. The editing efficiency of NHEJ and PAINT methods were analyzed by the QX200 Droplet Digital PCR (ddPCR) System (Bio-Rad). Primers and probes are listed in Supplementary Table S3.

### Statistical analysis

All statistical analyses (unless stated otherwise) were performed using the R package for Statistical Computing. For experimental data quantification, unpaired two-sided Student’ *t*-test was applied using GraphPad Prism 6 software, and the error bar was shown based on SEM (unless stated otherwise). *P* < 0.05 was considered statistically significant.

## Supplementary information

Supplementary information

SUPPLEMENTARY Table S1

SUPPLEMENTARY Table S2

SUPPLEMENTARY Table S3
