# Supplementary material for: High-efficiency targeted transgene integration via primed micro-homologues
Source: Cell Discov. 2023 Jul 4;9:69. doi: 10.1038/s41421-023-00552-0 (PMC10319781; doi:10.1038/s41421-023-00552-0)
Supplement: Supplementary file 1 — Supplementary information [file 41421_2023_552_MOESM1_ESM.pdf]

High-efficiency targeted transgene integration via primed micro-homologues

Chenxin Wang[1, 2, 3, 4, 6], Sen Fang[1, 2, 3, 6], Yangcan Chen[1, 2, 3, 6], Na Tang[1, 2, 3, 4], Guanyi Jiao[1, 2, 3], Yanping Hu[1, 2, 3], Jing Li[1, 2, 3], Qingtong Shan[5], Xin Wang[1, 2, 3], Guihai Feng[1, 2, 3, 4], Qi Zhou[1, 2, 3, 4 *], Wei Li[1, 2, 3, 4, *]

[1]State Key Laboratory of Stem Cell and Reproductive Biology, Institute of Zoology, Chinese Academy of Sciences, Beijing 100101, China

[2]Institute for Stem Cell and Regenerative Medicine, Chinese Academy of Sciences, Beijing 100101, China

[3]University of Chinese Academy of Sciences, Beijing 100049, China

[4]Bejing Institute for Stem Cell and Regenerative Medicine, Beijing 100101, China

[5]Northeast Agricultural University, Harbin, China

[6]These authors contributed equally: Chenxin Wang, Sen Fang, Yangcan Chen.

[*]These authors jointly supervised this work: Qi Zhou, Wei Li. ✉Email: zhouqi@ioz.ac.cn; liwei@ioz.ac.cn

Supplementary Information

Supplementary figures and figure legends

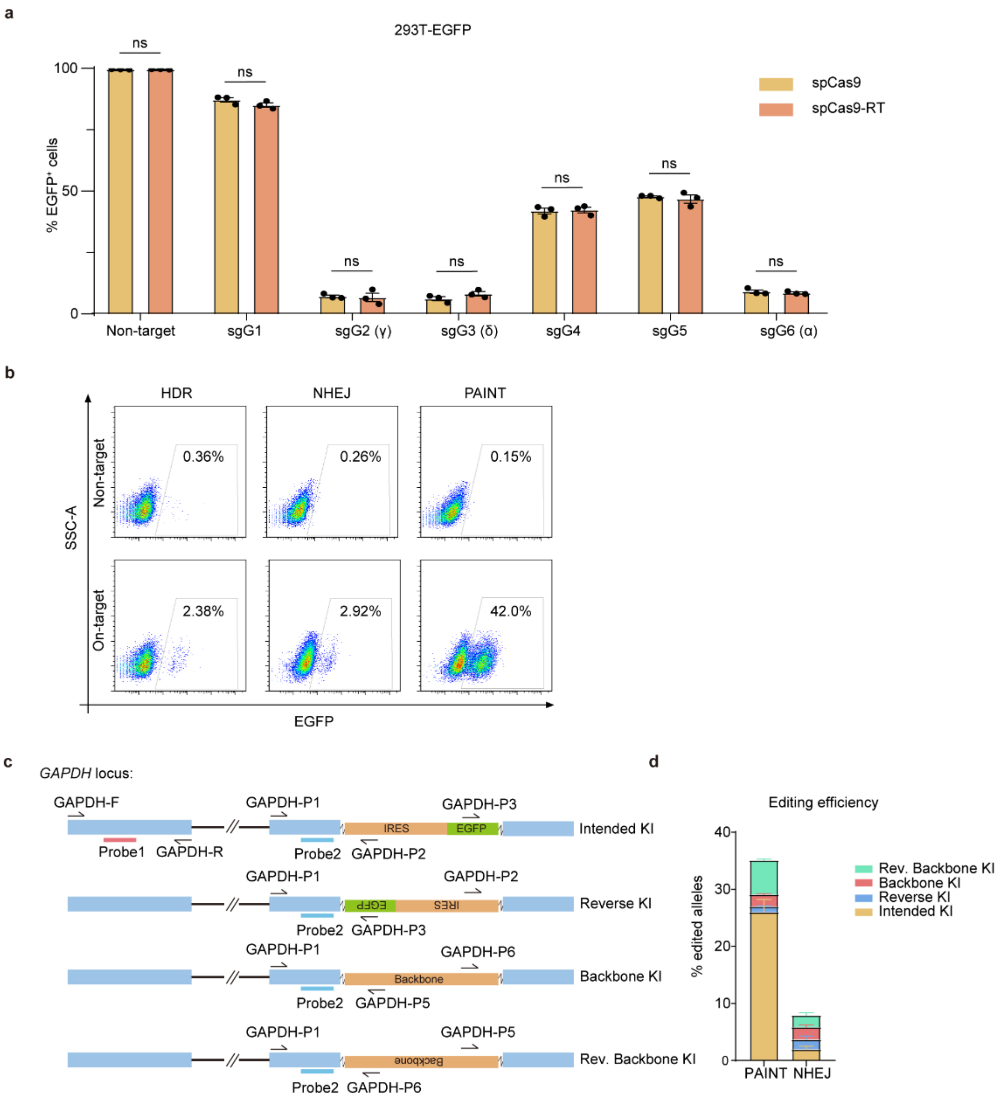

Supplementary Fig. S1 Primed micro-homologous overhangs induce highly efficient targeted transgene integrations. a Cleavage efficiencies of spCas9 and spCas9-RT at various loci in 293T-EGFP cells measured as EGFP$^+$ cells among total transfected cells. Three replicates were performed. The results are presented as the mean ± SEM. ns, no significance, unpaired Student's t-test, two-sided. **b** Scatter plots show EGFP$^+$ cell frequencies of HDR-, NHEJ-, and PAINT-mediated IRES-EGFP integration at the 3'-UTR of *GAPDH*. **c** Diagram

shows NHEJ- and PAINT-mediated intended editing and on-target integration errors (reverse KIs and backbone KIs) at the *GAPDH* target site. Positions of primers and probes used for droplet digital PCR (ddPCR) are shown as arrows. **d** Editing efficiencies of NHEJ- and PAINT-mediated IRES-EGFP integration at the 3'-UTR of *GAPDH* measured by ddPCR. The portions of edited alleles with intended KI, reverse KI, and backbone KIs were analyzed. Three replicates were performed. The results are presented as the mean ± SEM.

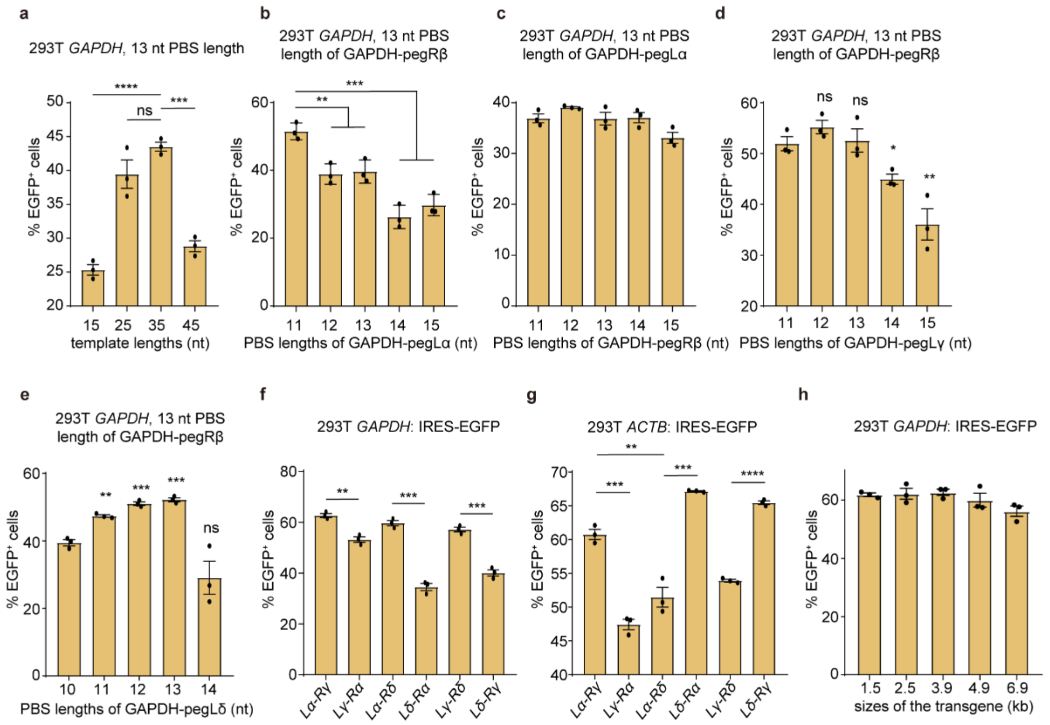

Supplementary Fig. S2 Optimization for parameters of PAINT-mediated transgene integration. a Editing efficiencies of PAINT-mediated IRES-EGFP integration at the 3'-UTR of *GAPDH* with varying pegRNA RT-template lengths. **b** Editing efficiencies of PAINT-mediated IRES-EGFP integration at the 3'-UTR of *GAPDH* with varying PBS lengths of pegLα. **c** Editing efficiencies of PAINT-mediated IRES-EGFP integration at the 3'-UTR of *GAPDH* with varying PBS lengths of pegRβ. **d** Editing efficiencies of PAINT-mediated IRES-EGFP integration at the 3'-UTR of *GAPDH* with varying PBS lengths of pegLγ. **e** Editing efficiencies of PAINT-mediated IRES-EGFP integration at the 3'-UTR of *GAPDH* with varying PBS lengths of pegLδ. **f** Editing efficiencies of PAINT-mediated IRES-EGFP integration at the 3'-UTR of *GAPDH* with various pegRNA pairs. **g** Editing efficiencies of PAINT-mediated IRES-EGFP integration at the 3'-UTR of *ACTB* with various pegRNA pairs. **h** Editing efficiencies of PAINT-mediated IRES-EGFP integration at the 3'-UTR of *GAPDH* with transgenes of different sizes.

Editing efficiencies were measured as the percentage of $EGFP^+$ cells among total transfected cells. Three replicates were used. The results are presented as the mean ± SEM. ns, no significance; *$p < 0.05$, **$p < 0.01$, ***$p < 0.001$, ****$p < 0.0001$, unpaired Student’s t-test, two-sided.

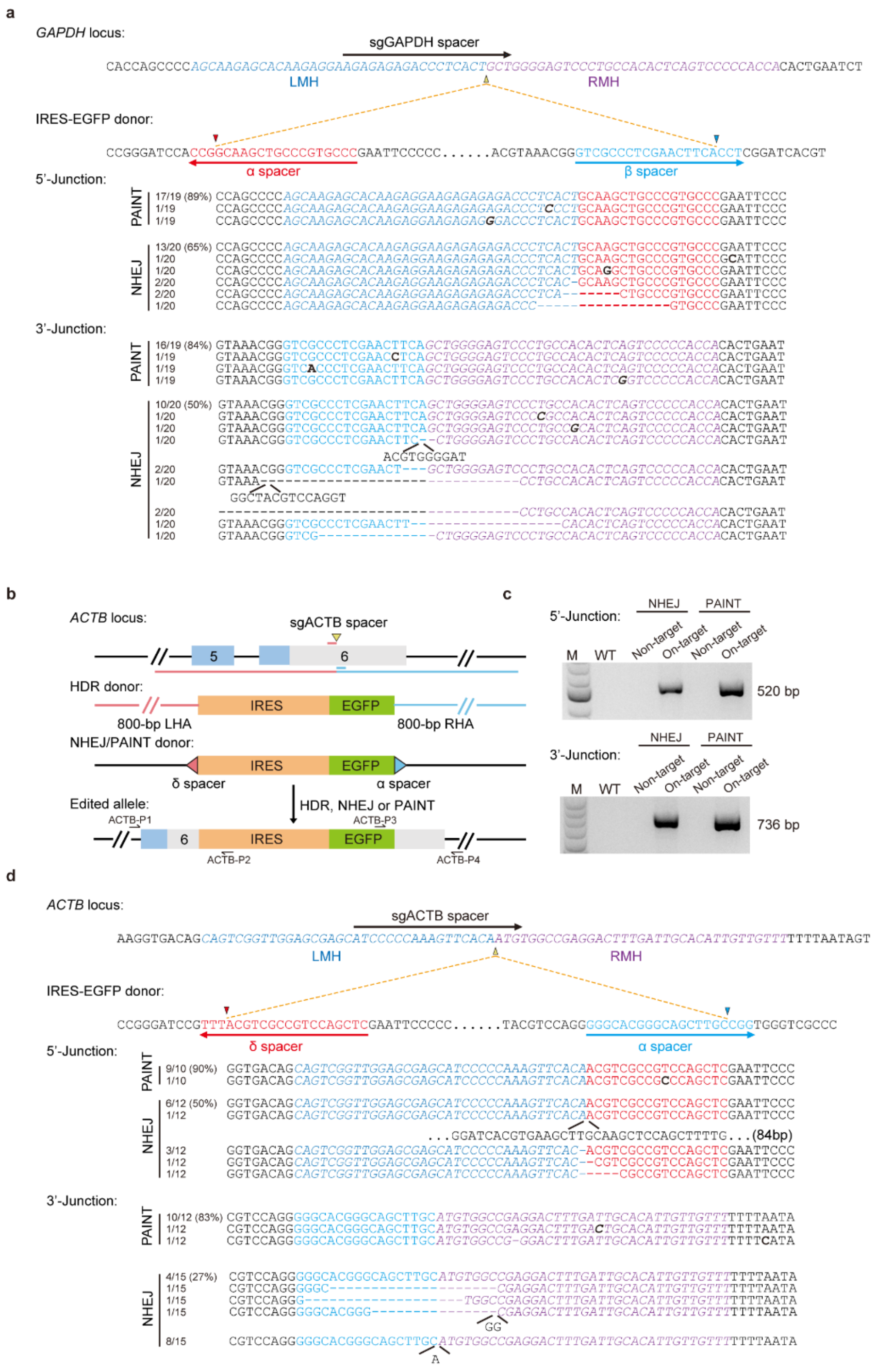
a
GAPDH locus:
sgGAPDH spacer
LMH
RMH
IRES-EGFP donor:
α spacer
β spacer
5'-Junction:
PAINT
NHEJ
3'-Junction:
PAINT
NHEJ
b
ACTB locus:
sgACTB spacer
5
6
HDR donor:
IRES
EGFP
800-bp LHA
800-bp RHA
NHEJ/PAINT donor:
δ spacer
α spacer
Edited allele:
HDR, NHEJ or PAINT
ACTB-P1
ACTB-P2
ACTB-P3
ACTB-P4
c
5'-Junction:
NHEJ
PAINT
M
WT
Non-target
On-target
520 bp
3'-Junction:
736 bp
d
ACTB locus:
sgACTB spacer
LMH
RMH
IRES-EGFP donor:
δ spacer
α spacer
5'-Junction:
PAINT
NHEJ
3'-Junction:
PAINT
NHEJ

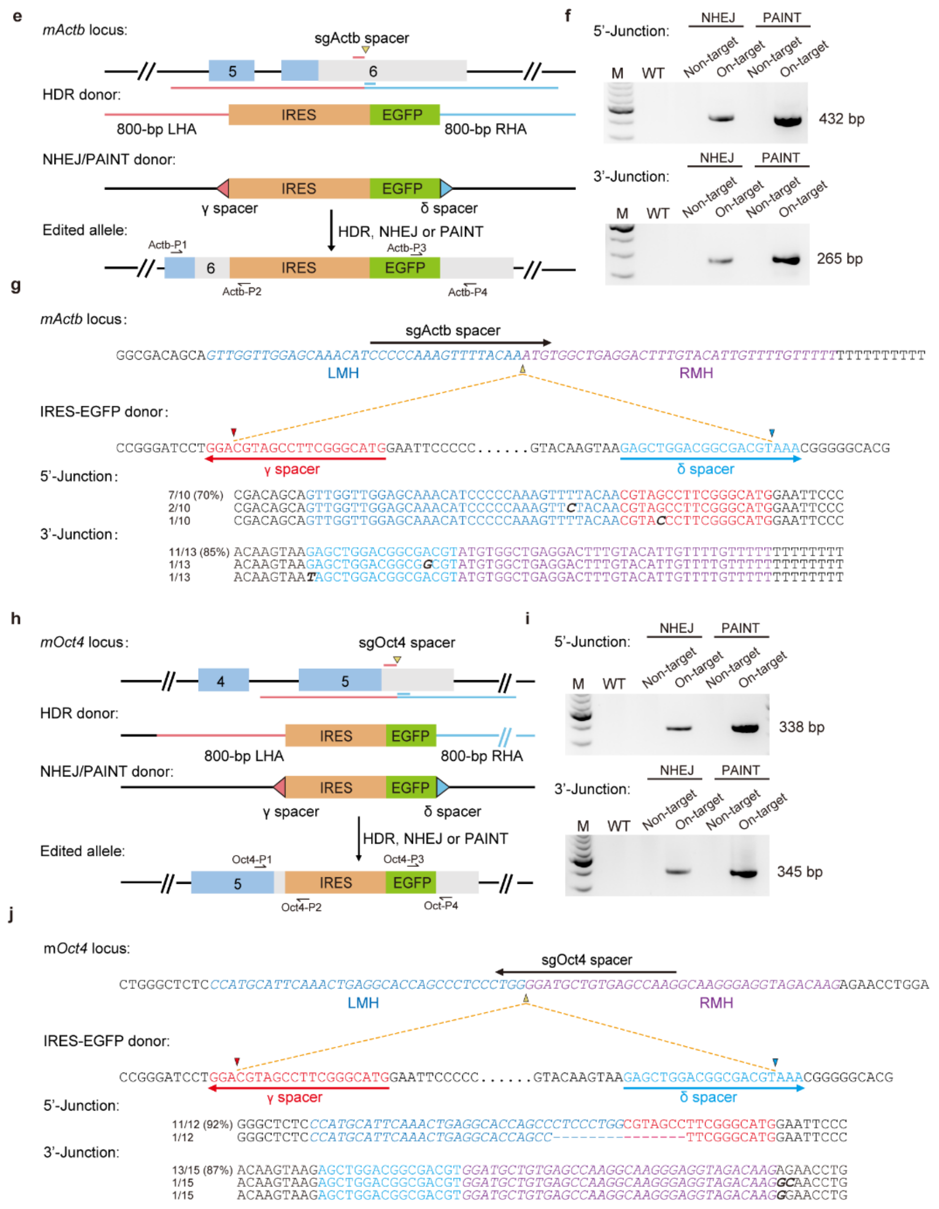

Supplementary Fig. S3 Genotyping analysis of PAINT-mediated IRES-EGFP integration at various genomic loci across different cell types. **a** Sanger sequencing of junctions of NHEJ- and PAINT-mediated IRES-EGFP integration at the 3'-UTR of *GAPDH*. Triangles indicate cleavage sites in the genomic DNA and exogenous donor. LMH, left micro-homologue; RMH, right micro-homologue. **b** Diagram shows HDR-, NHEJ-, and PAINT-mediated IRES-EGFP integration at the 3'-UTR of *ACTB*. The yellow triangle marks the target site recognized by sgACTB. The long red and cyan lines indicate the left and right HAs, respectively. The short

red and cyan lines indicate the left and right HMs, respectively. The red and cyan triangles indicate the generic spaces targeted by sgδ (or pegδ) and sgα (or pegα), respectively. The pegRNAs were designed according to the MHs and spacers. Positions of primers for PCR genotyping of correctly edited alleles are shown as arrows. **c** PCR genotyping of NHEJ- and PAINT-mediated IRES-EGFP integration at the 3'-UTR of *ACTB*. Primers ACTB-P1/ACTB-P2 and ACTB-P3/ACTB-P4 amplify the 5' junction (520 bp) and the 3' junction (736 bp) on correctly edited *ACTB* alleles, respectively. **d** Sanger sequencing of junctions of NHEJ- and PAINT-mediated IRES-EGFP integration at the 3'-UTR of *ACTB*. Triangles indicate the cleavage sites in the genomic DNA and exogenous donor. LMH, left micro-homologue; RMH, right micro-homologue. **e** Diagram shows HDR-, NHEJ-, and PAINT-mediated IRES-EGFP integration at the 3'-UTR of mouse *Actb* gene. The yellow triangle marks the genomic target site recognized by sgActb at the 3'-UTR of *Actb*. The long red and cyan lines indicate the left and right HAs, respectively. The short red and cyan lines indicate the left and right MHs, respectively. The red and cyan triangles indicate the generic spacers targeted by sgγ (or pegγ) and sgδ (or pegδ), respectively. The pegRNAs were designed according to the MHs and spacers. Positions of primers for PCR genotyping of correctly edited alleles are shown as arrows. **f** PCR genotyping of NHEJ- and PAINT-mediated IRES-EGFP integration at the 3'-UTR of *Actb*. Primers Actb-P1/Actb-P2 and Actb-P3/Actb-P4 amplify the 5' junction (432 bp) and the 3' junction (265 bp) on correctly edited *Actb* alleles, respectively. **g** Sanger sequencing of the NHEJ- and PAINT-mediated IRES-EGFP integration junctions at the 3'-UTR of *Actb*. Triangles indicate the cleavage sites in the genomic DNA and exogenous donor. LMH, left micro-homologue; RMH, right micro-homologue. **h** Diagram shows HDR-, NHEJ-, and PAINT-

mediated IRES-EGFP integration at the 3'-UTR of mouse *Oct4* gene. The yellow triangle marks the genomic target site recognized by sgOct4 at the *Oct4* 3'-UTR. The long red and cyan lines indicate the left and right HAs, respectively. The short red and cyan lines indicate the left and right MHs, respectively. The red and cyan triangles indicate the generic spacers targeted by sgγ (or pegγ) and sgδ (or pegδ), respectively. Positions of primers for PCR genotyping of correctly edited alleles are shown as arrows. **i** PCR genotyping of NHEJ- and PAINT-mediated IRES-EGFP integration at the 3'-UTR of *Oct4*. Primers Oct4-P1/Oct4-P2 and Oct4-P3/Oct4-P4 amplify the 5' junction (338 bp) and the 3' junction (345 bp) on correctly edited *Oct4* alleles, respectively. **j** Sanger sequencing for the junctions of NHEJ- and PAINT-mediated IRES-EGFP integration at the 3'-UTR of *Oct4*. Triangles indicate the cleavage sites in the genomic DNA and exogenous donor. LMH, left micro-homologue; RMH, right micro-homologue.

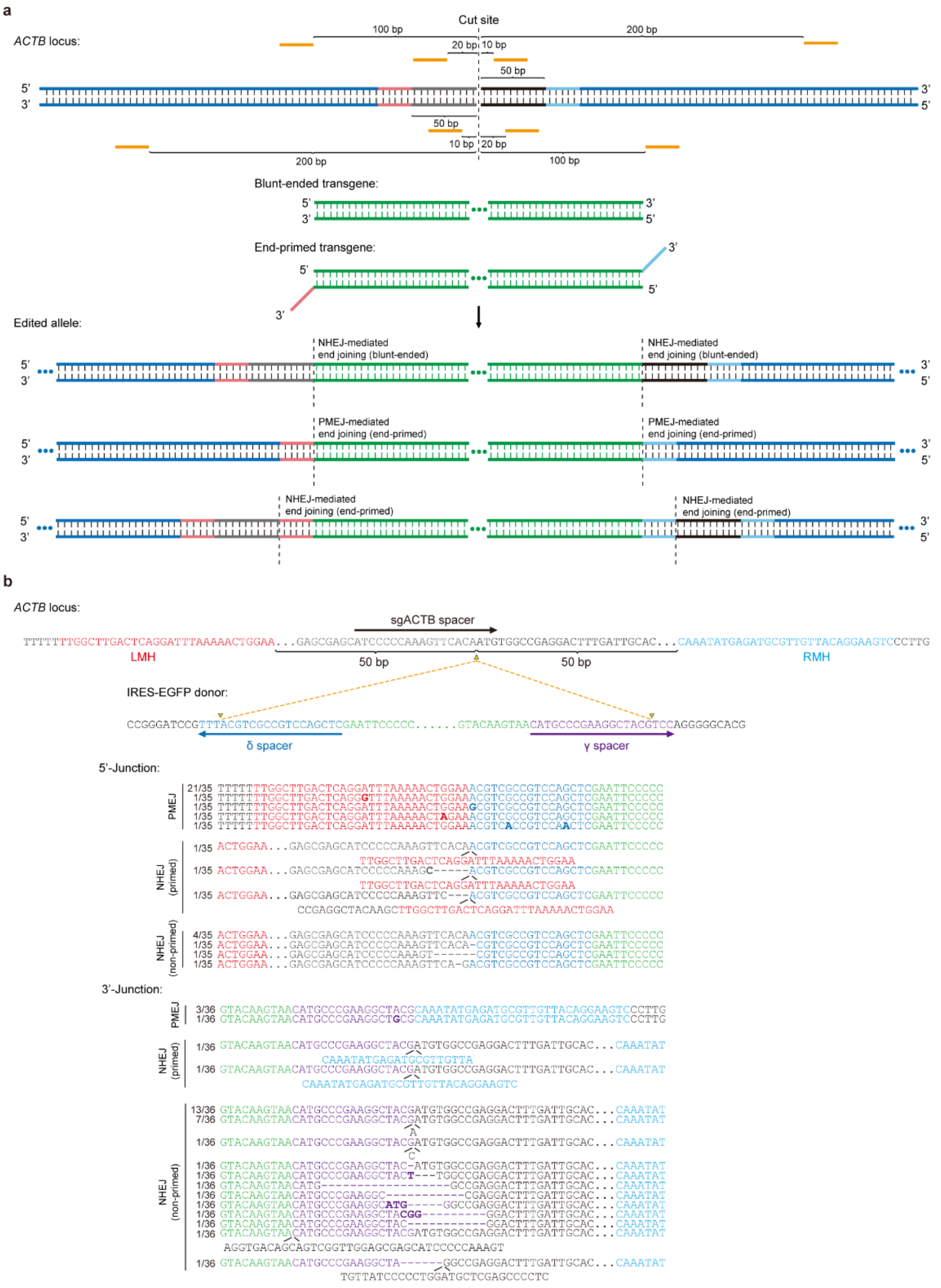

Supplementary Fig. S4 PAINT-mediated targeted integration with inner MHs. a Diagram shows PAINT-mediated IRES-EGFP integration at the 3'-UTR of human *ACTB* gene with inner MHs that leave a distance between the cut site and the intended integration (MH) site. Each line represents a DNA strand. Genomic DNA is marked in blue except that the inner MHs (LMH and RMH) 50 bp away from the cut site are marked in red and cyan, respectively, and that the

spacers between the 50-bp MHs and the cut site are marked in gray and black, respectively. Positions of inner MHs with other spacer lengths are marked with yellow short lines. The transgene is marked in green. In the PAINT system, both blunt-ended and end-primed transgenes are present. For PAINT-mediated targeted KI with inner MHs, blunt-ended transgene incorporates into the genome at the cut site via NHEJ. While the end-primed transgene may either incorporates into the genome at the MH site via primed micro-homologue-mediated end joining (PMEJ) or at the cut site via NHEJ. **b** Sanger sequencing analysis of amplified edited junctions (primers ACTB-P1/ ACTB P2 and ACTB-P3/ACTB-P4) detects both NHEJ- and PMEJ-based transgene integration in PAINT-mediated targeted KI with inner MHs at the 3'-UTR of human *ACTB* gene. Both LMH and RMH are 50 bp away from the cut site. Triangles indicate the cleavage sites in the genomic DNA and exogenous donor. LHM, left micro-homologue; RHM, right micro-homologue.

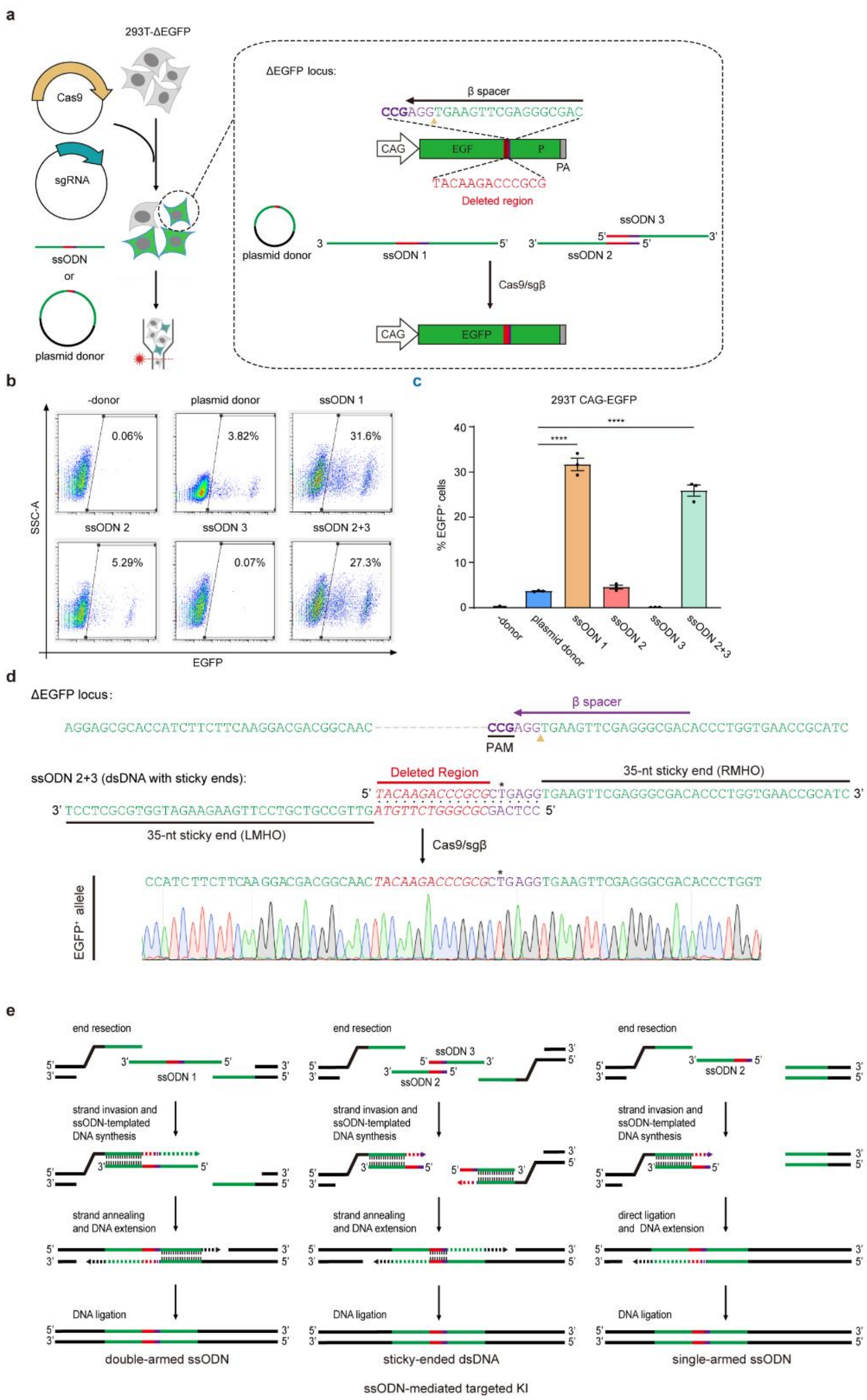

Supplementary Fig. S5 Recovery of EGFP expression with sticky-ended dsDNA template in the 293T-ΔEGFP cell line. a Schematic diagram shows the recovery of a CAG promoted EGFP-expressing gene in 293T-ΔEGFP cells with a plasmid donor or short DNA repair templates. ΔEGFP carry a 13-bp deletion in the coding region that depleted EGFP expression. Correctly edited cells express EGFP and the editing efficiency can be measured by detection of

the frequency of EGFP$^+$ cells by FACS. The plasmid donor harbors 500-bp long left and right HAs. ssODNs have 35-nt MHs on both sides (ssODN 1) or on either side (ssODN 2 and ssODN 3). Strips and lines represent double-stranded DNAs and single-stranded DNAs, respectively. The EGFP gene is marked in green, except that the deleted region is marked in red, and that the spacer region between the deleted region and the cut site is marked in purple. The cut site is recognized by Cas9/sgβ. PA, ploy A signal site. **b** Scatter plots show editing efficiencies for the recovery of the mutant EGFP gene in 293T-ΔEGFP cells with a plasmid donor, a double-armed ssODN (ssODN 1), a single-armed ssODNs (ssODN 1 and ssODN 2) and a sticky-ended dsDNA (by annealing ssODN 2 with ssODN 3). FACS demonstrates that both the double-armed ssODN and the sticky-ended dsDNA manifest high editing efficacies. **c** Bar chats for statistics of the editing efficiencies for the recovery of the mutant EGFP gene in 293T-ΔEGFP cells with the plasmid donor or short DNA repair templates of various forms. Gene recovery efficiencies were measured as the percentage of EGFP$^+$ cells among total transfected cells. Three replicates were performed. The results are presented as the mean ± SEM. ns, no significance, ****$p < 0.0001$, unpaired Student's t-test, two-sided. **d** Sanger sequencing confirms precise gene correction with the sticky-ended dsDNA donor. Nonsense PAM mutation is marked with an asterisk. **e** Schematic diagram shows a synthesis-dependent strand annealing (SDSA) model in double-armed ssODN- and sticky-ended dsDNA-mediated targeted integration. Each line corresponds to a DNA strand. HAs are marked in green, the edit is marked in red, the spacer region between the edit site and the cut site is marked in purple. The comb teethes represent the annealing of homologous strands. For double-armed ssODN, the donor first invades the 3'-end of the short-range resected genomic DNA with its 3'-HA and initiates

ssODN-templated DNA repair synthesis. The synthesized homologue of 5'-HA then pairs with the other homologous 3'-DNA end to mediate rejoining of genomic DNA. The edit is integrated into the targeted site during the ssODN-templated DNA synthesis process. For sticky-ended dsDNA, each strand of the dsDNA invades the corresponding short-range resected genomic DNA with its 3'-HA and dictates DNA repair synthesis. The two synthesized homologous 3'-DNA end (each including the edit and the spacer region) then anneal to each other to mediate rejoining of cleaved genomic DNA. For single-armed ssODN, the ssODN first invades the 3'-end of the resected genomic DNA with its 3'-HA and dictates DNA synthesis. The two genomic DNA ends then directly rejoin together via NHEJ. Both double-armed ssODN and sticky-ended dsDNA mediate targeted integration via SDSA with single-stranded 3'-MHs. During the strand invasion process, each MH pairs with the homologous genomic DNA end independent of extensive genomic DNA end resection.

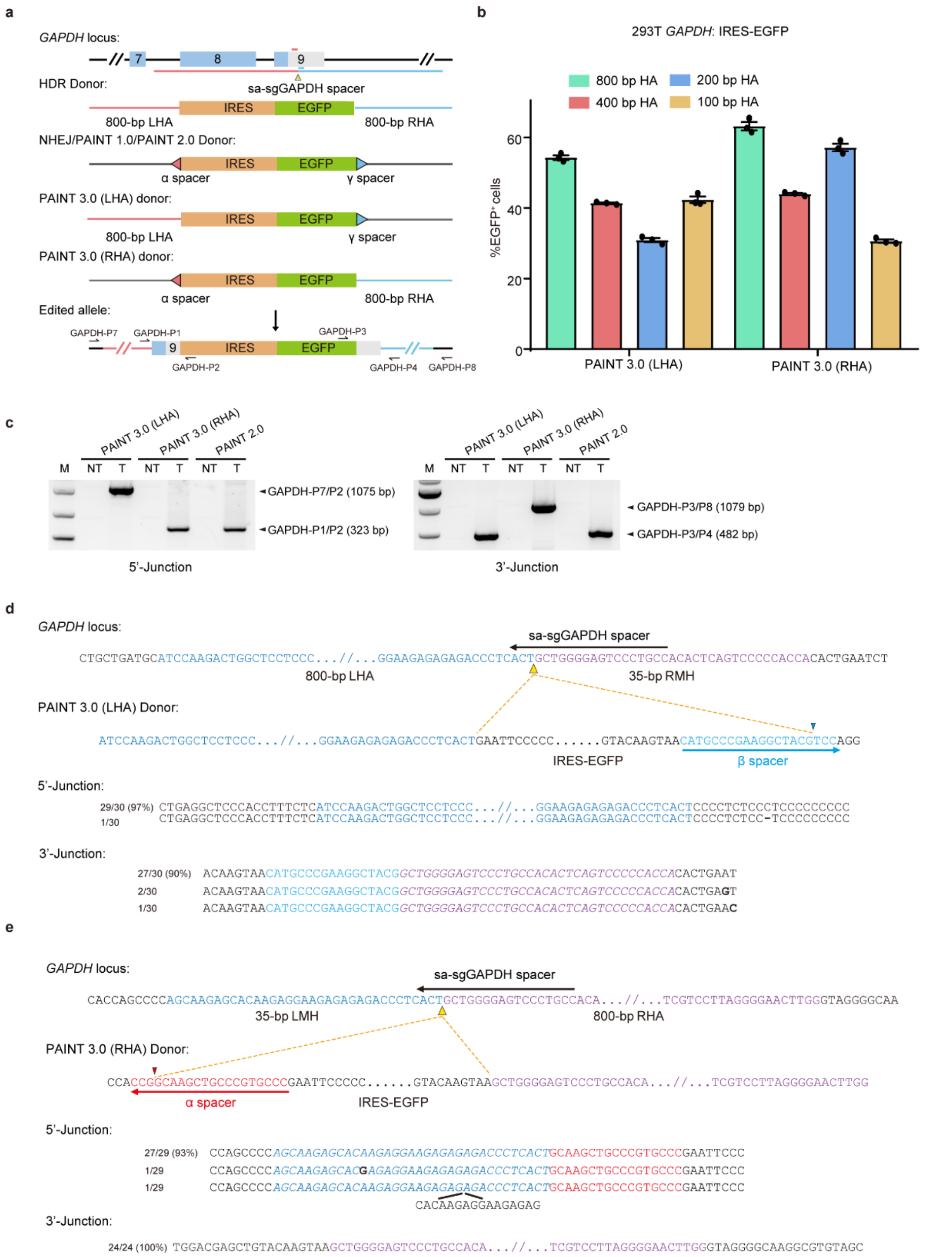

Supplementary Fig. S6 PAINT 3.0 mediated IRES-EGFP transgene integration at the 3'-UTR of *GAPDH*. a Diagram shows HDR-, NHEJ-, and PAINT 1.0-, PAINT 2.0-, and PAINT 3.0-mediated IRES-EGFP transgene integrations at the 3'-UTR of *GAPDH*. The yellow triangle marks the genomic target site recognized by sa-sgGAPDH. The long red and cyan lines indicate

the 800-bp left and right HAs, respectively. The short red and cyan lines indicate the 35-bp left and right MHs, respectively. The red and cyan triangles indicate the generic spacers targeted by sgα (or pegα) and sgγ (or pegγ), respectively. PegRNAs were designed corresponding to the MHs and spacers. Positions of primers for PCR genotyping are shown as arrows. **b** Editing efficiencies of PAINT 3.0-mediated IRES-EGFP integration at the 3'-UTR of *GAPDH* with PAINT 3.0 donors of varying HA lengths. **c** PCR genotyping of PAINT 3.0-mediated IRES-EGFP integration at the 3'-UTR of *GAPDH.* For the PAINT 3.0 (LHA) system, primers GAPDH-P7/GAPDH-P2 and GAPDH-P3/GAPDH-P4 amplify the 5' junction (1075 bp) and the 3' junction (482 bp) on correctly edited *GAPDH* alleles, respectively. For the PAINT 3.0 (RHA) system, primers GAPDH-P1/GAPDH-P2 and GAPDH-P3/GAPDH-P8 amplify the 5' junction (323 bp) and the 3' junction (1079 bp) on correctly edited *GAPDH* alleles, respectively. **d** Sanger sequencing for the junctions of PAINT 3.0 (LHA)-mediated IRES-EGFP integration at the 3'-UTR of *GAPDH.* Triangles indicate the cleavage sites in the genomic DNA and exogenous donor. LHA, left homologues arm; RMH, right micro-homologue. **e** Sanger sequencing for junctions of PAINT 3.0 (RHA)-mediated IRES-EGFP integration at the 3'-UTR of *GAPDH.* Triangles indicate the cleavage sites in the genomic DNA and exogenous donor. LHM, left micro-homologue; RHA, right homologues arm.

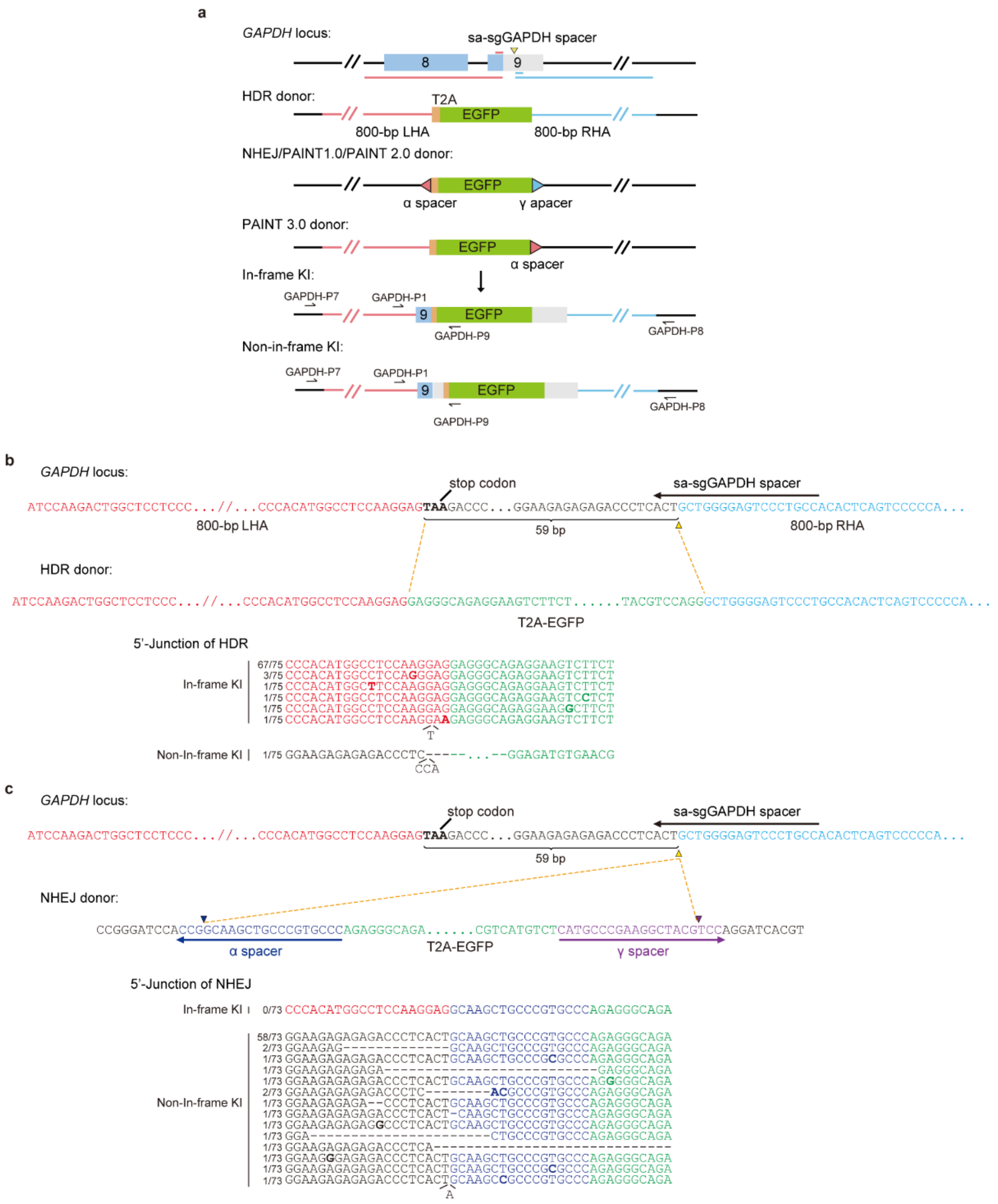
a
GAPDH locus:
sa-sgGAPDH spacer
8
9
HDR donor:
T2A
EGFP
800-bp LHA
800-bp RHA
NHEJ/PAINT1.0/PAINT 2.0 donor:
EGFP
α spacer
γ apacer
PAINT 3.0 donor:
EGFP
α spacer
In-frame KI:
GAPDH-P7
GAPDH-P1
9
EGFP
GAPDH-P9
GAPDH-P8
Non-in-frame KI:
GAPDH-P7
GAPDH-P1
9
EGFP
GAPDH-P9
GAPDH-P8
b
GAPDH locus:
stop codon
sa-sgGAPDH spacer
ATCCAAGACTGGCTCCTCCC...//...CCCACATGGCCTCCAAGGAGTAAGACCC...GGAAGAGAGAGACCCTCACTGCTGGGGAGTCCCTGCCACACTCAGTCCCCCA...
800-bp LHA
59 bp
800-bp RHA
HDR donor:
ATCCAAGACTGGCTCCTCCC...//...CCCACATGGCCTCCAAGGAGGAGGGCAGAGGAAGTCTTCT......TACGTCCAGGGCTGGGGAGTCCCTGCCACACTCAGTCCCCCA...
T2A-EGFP
5'-Junction of HDR
In-frame KI
67/75 CCCACATGGCCTCCAAGGAGGAGGGCAGAGGAAGTCTTCT
3/75 CCCACATGGCCTCCAGGGAGGAGGGCAGAGGAAGTCTTCT
1/75 CCCACATGGCTTCCAAGGAGGAGGGCAGAGGAAGTCTTCT
1/75 CCCACATGGCCTCCAAGGAGGAGGGCAGAGGAAGTCCTCT
1/75 CCCACATGGCCTCCAAGGAGGAGGGCAGAGGAAGGCTTCT
1/75 CCCACATGGCCTCCAAGGAAGAGGGCAGAGGAAGTCTTCT
T
Non-In-frame KI
1/75 GGAAGAGAGAGACCCTC-----...--GGAGATGTGAACG
CCA
c
GAPDH locus:
stop codon
sa-sgGAPDH spacer
ATCCAAGACTGGCTCCTCCC...//...CCCACATGGCCTCCAAGGAGTAAGACCC...GGAAGAGAGAGACCCTCACTGCTGGGGAGTCCCTGCCACACTCAGTCCCCCA...
59 bp
NHEJ donor:
CCGGGATCCACCGGCAAGCTGCCCGTGCCCAGAGGGCAGA......CGTCATGTCTCATGCCCGAAGGCTACGTCCAGGATCACGT
α spacer
T2A-EGFP
γ spacer
5'-Junction of NHEJ
In-frame KI
0/73 CCCACATGGCCTCCAAGGAGGCAAGCTGCCCGTGCCCAGAGGGCAGA
Non-In-frame KI
58/73 GGAAGAGAGAGACCCTCACTGCAAGCTGCCCGTGCCCAGAGGGCAGA
2/73 GGAAGAG-------------GCAAGCTGCCCGTGCCCAGAGGGCAGA
1/73 GGAAGAGAGAGACCCTCACTGCAAGCTGCCCGCGCCCAGAGGGCAGA
1/73 GGAAGAGAGAGA------------------------GAGGGCAGA
1/73 GGAAGAGAGAGACCCTCACTGCAAGCTGCCCGTGCCCAGGGGGCAGA
2/73 GGAAGAGAGAGACCCTC--------ACGCCCGTGCCCAGAGGGCAGA
1/73 GGAAGAGAGA--CCCTCACTGCAAGCTGCCCGTGCCCAGAGGGCAGA
1/73 GGAAGAGAGAGACCCTCACT-CAAGCTGCCCGTGCCCAGAGGGCAGA
1/73 GGAAGAGAGAGGCCCTCACTGCAAGCTGCCCGTGCCCAGAGGGCAGA
1/73 GGA----------------------CTGCCCGTGCCCAGAGGGCAGA
1/73 GGAAGAGAGAGACCCTCA-----------------------------
1/73 GGAAGGGAGAGACCCTCACTGCAAGCTGCCCGTGCCCAGAGGGCAGA
1/73 GGAAGAGAGAGACCCTCACTGCAAGCTGCCCGCGCCCAGAGGGCAGA
1/73 GGAAGAGAGAGACCCTCACTGCAAGCCGCCCGTGCCCAGAGGGCAGA
A

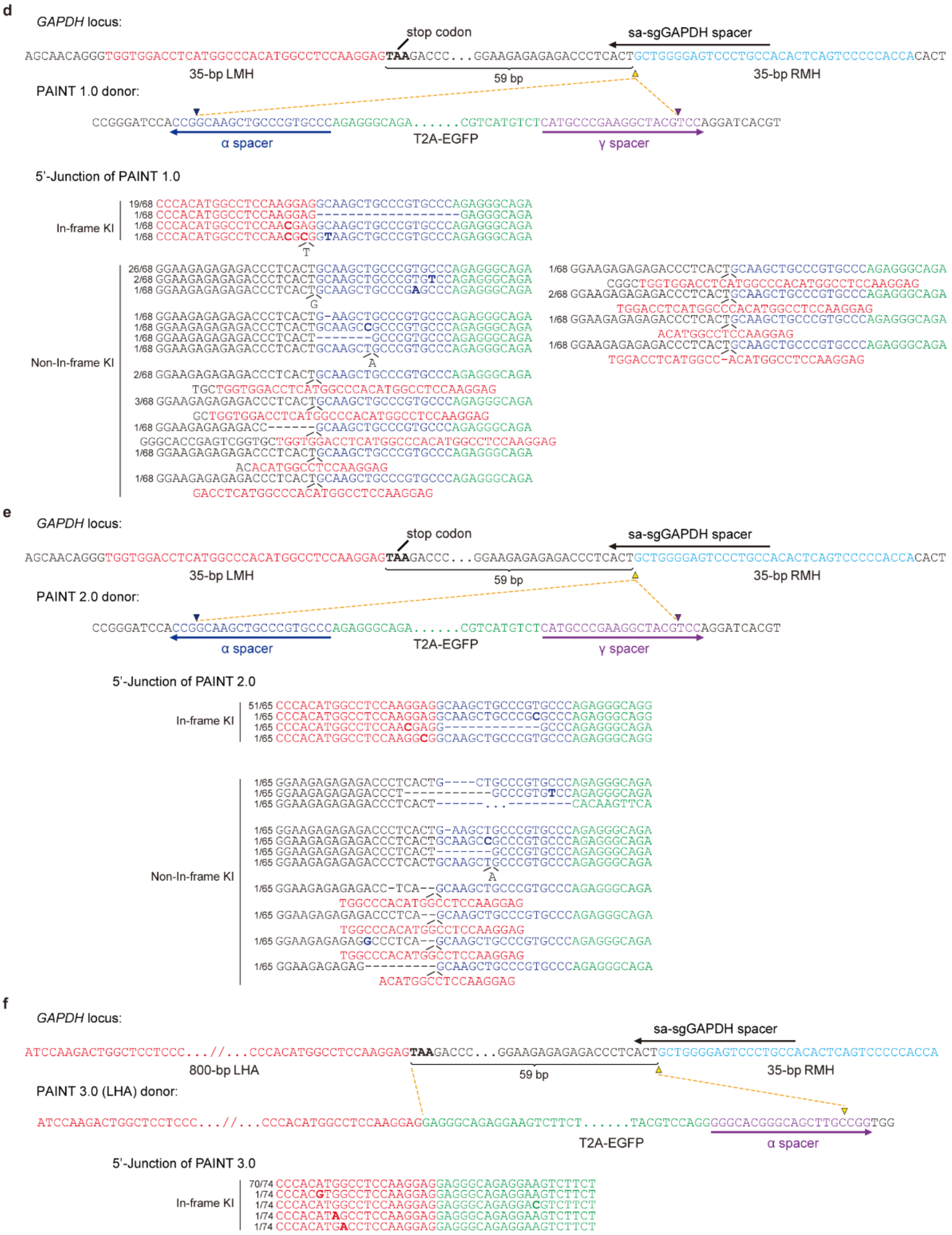

Supplementary Fig. S7 PAINT 3.0 mediates in-frame T2A-EGFP transgene integration at the *GAPDH* locus. a Diagram shows HDR-, NHEJ-, and PAINT 1.0-, PAINT 2.0-, and PAINT 3.0-mediated T2A-EGFP transgene integration at the 3'-UTR of *GAPDH*. The yellow triangle marks the genomic target site recognized by sa-sgGAPDH. The long red and cyan lines indicate the 800-bp left and right HAs, respectively. The short red and cyan lines indicate the

35-bp left and right MHs, respectively. The red and cyan triangles indicate the generic spacers targeted by sgα (or pegα) and sgγ (or pegγ), respectively. PegRNAs were designed corresponding to the MHs and spacers. Positions of primers for PCR genotyping are shown as arrows. **b** Sanger sequencing analysis of the amplified 5'-junction in HDR-based targeted integration of the T2A-EGFP transgene detects edited alleles with in-frame transgene KIs right upstream of the stop codon and a very small portion of edited alleles with non-in-frame transgene KIs at the cut site. **c** Sanger sequencing analysis of the amplified 5'-junction in NHEJ-based targeted integration of the T2A-EGFP transgene detects only edited alleles with non-in-frame transgene KIs at the cut site. **d** Sanger sequencing analysis of the amplified 5'-junction in PAINT 1.0-based targeted integration of the T2A-EGFP transgene detects a small portion of edited alleles with in-frame transgene KIs right upstream of the stop codon and a large portion of edited alleles with non-in-frame transgene KIs at the cut site. **e** Sanger sequencing analysis of the amplified 5'-junction in PAINT 2.0-based targeted integration of the T2A-EGFP transgene detects a large portion of edited alleles with in-frame transgene KIs right upstream of the stop codon and a small portion of edited alleles with non-in-frame transgene KIs at the cut site. **f** Sanger sequencing analysis of the amplified 5'-junction in PAINT 3.0-based targeted integration of the T2A-EGFP transgene detects only edited alleles with in-frame transgene KIs right upstream of the stop codon.

For **b**-**f**, triangles indicate the cleavage sites in the genomic DNA and exogenous donors. LHA, left homologous arm; RHA, right homologous arm; LHM, left micro-homologue; RHM, right micro-homologue.

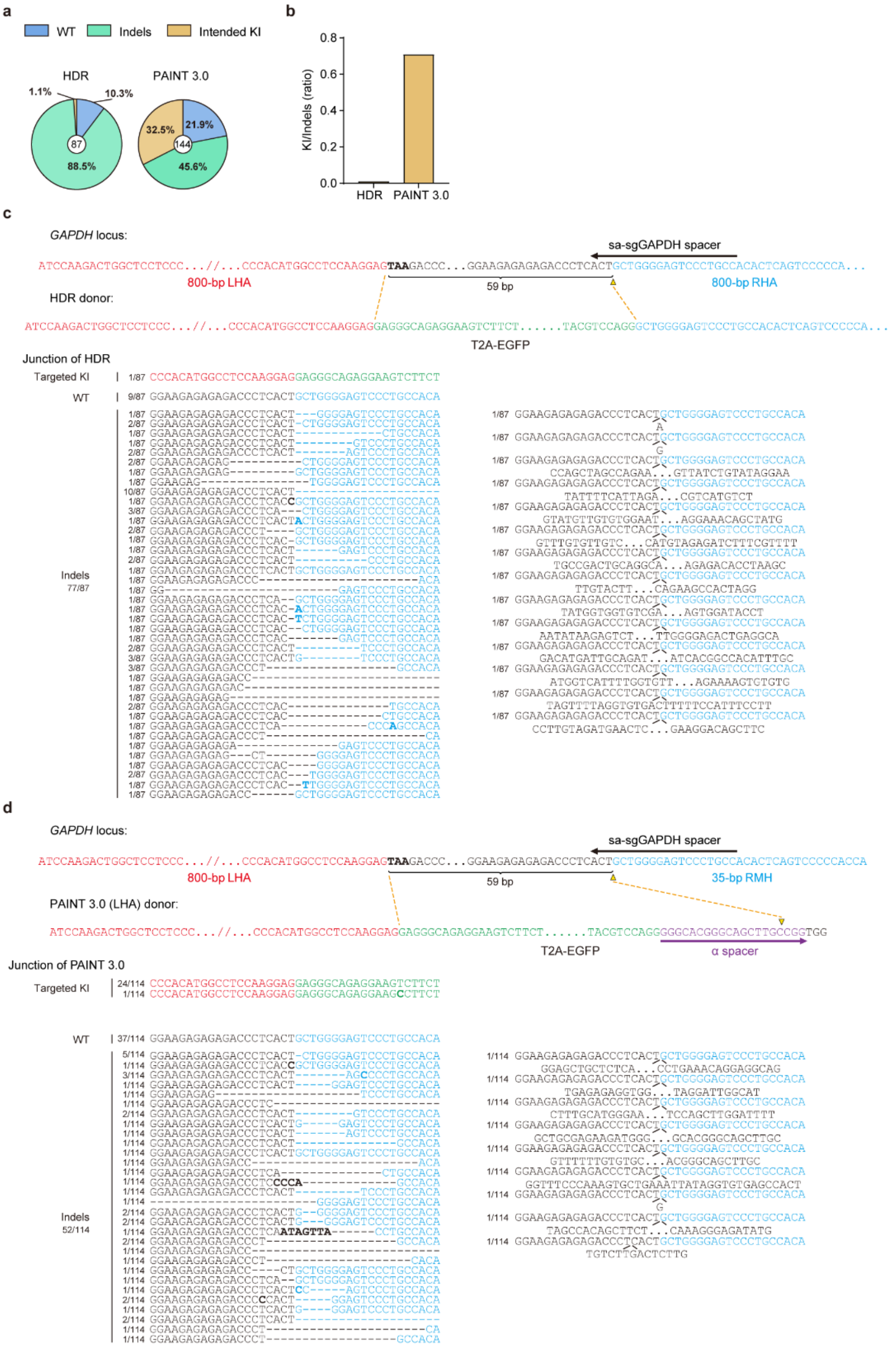

Supplementary Fig. S8 PAINT 3.0 mediates targeted transgene integration with reduced indels at the cut site. a Pie charts for statistics of Sanger sequencing analysis show portions of WT alleles, alleles with indels and alleles with targeted KI in HDR- and PAINT 3.0-mediated targeted integration of the T2A-EGFP transgene at the *GAPDH* locus. Primers GAPDH-

P7/GAPDH-P8 were used for PCR amplification, and GAPDH-P1 was used for Sanger sequencing. Compared with HDR, PAINT 3.0 achieves an increase ratio of targeted KI and reduced portion of alleles with indels. **b** Bar chart shows that PAINT 3.0 achieves increased KI/indels ratio compared with HDR. **c** Sanger sequencing analysis of the target genomic locus detects a small portion of in-frame transgene KI alleles and a large portion of alleles with indels in HDR-based targeted integration of the T2A-EGFP transgene. Wide type (WT) alleles are also shown. **d** Sanger sequencing analysis of the target genomic locus detects a large portion of in-frame transgene KI alleles and a relatively small portion of alleles with indels in PAINT 3.0-mediated targeted integration of the T2A-EGFP transgene. WT alleles are also shown.

For **c** and **d**, triangles indicate the cleavage sites in the genomic DNA and exogenous donors. LHA, left homologous arm; RHA, right homologous arm; RHM, right micro-homologue.

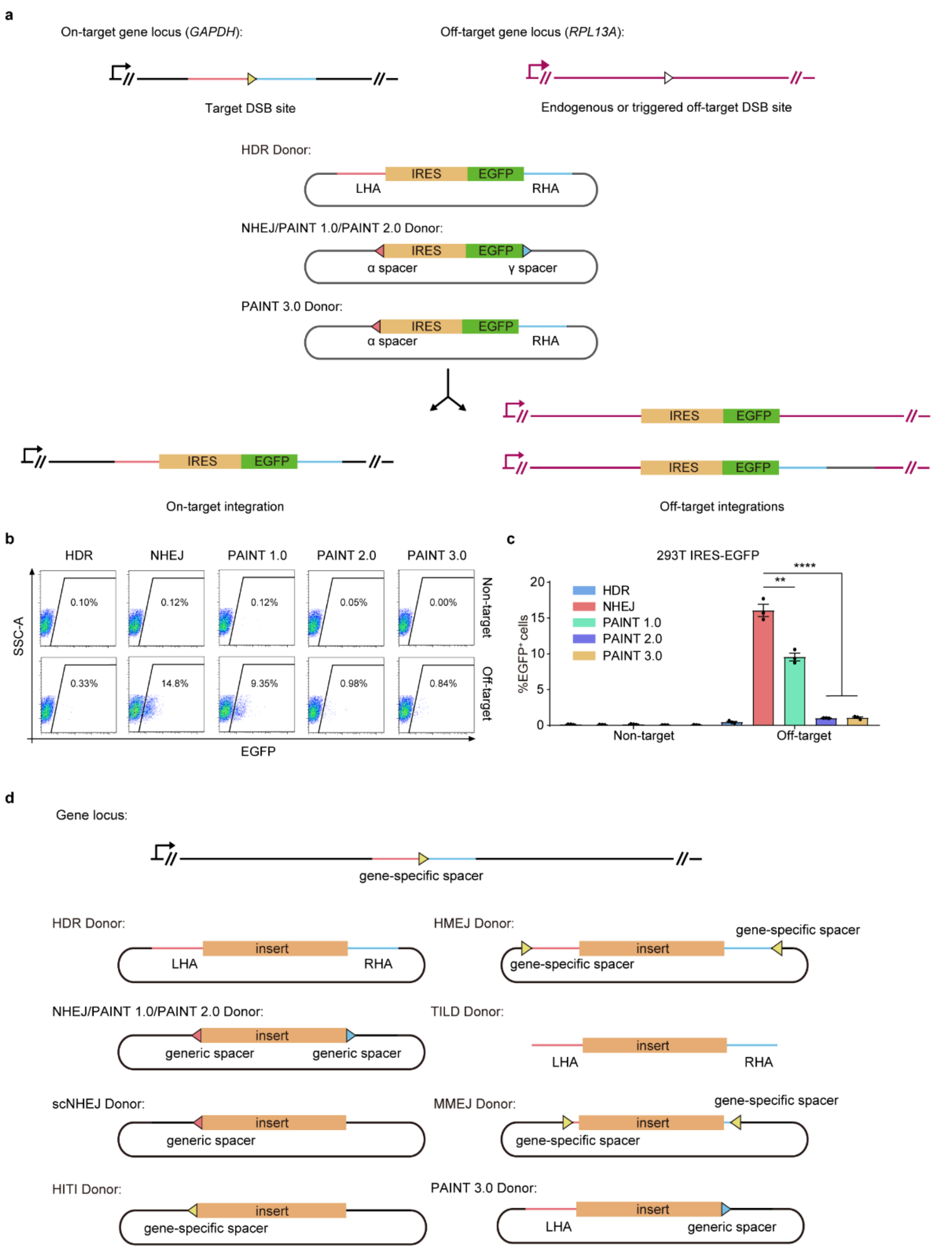

Supplementary Fig. S9 Primed micro-homologous flaps minimize unintended transgene integrations at off-target sites. a Diagram shows HDR-, NHEJ-, PAINT 1.0-, PAINT 2.0-, and PAINT 3.0 induced off-target IRES-EGFP transgene integrations at endogenous or programable nuclease triggered off-target DSB sites. **b** FACS scatter plots show off-target KI efficiencies by various methods at a triggered off-target DSB site (*RPL13A*). **c** Off-target KI

efficiencies at a triggered off-target DSB site (*RPL13A*) shows that both PAINT 2.0 and PAINT 3.0 achieves minimized off-target transgene integrations compared to that of NHEJ and PAINT 1.0. **d** Diagram shows various methods for targeted transgene KI. The HDR-, HMEJ-, and TILD-donors harbor long range HAs flanking the transgene cassette. The MMEJ-donor harbors MHs of 20 nt in length. scNHEJ- and HITI-donors are cleaved by a generic (scNHEJ) or gene-specific (HITI) targeting spacer. Both MMEJ- and HMEJ-donors are linearized by gene-specific spacers. The PAINT 3.0-donor harbors a single generic spacer and a double-stranded HA flanking the transgene cassette.

For **b-c**, editing efficiencies were measured as the percentage of $EGFP^+$ cells among total transfected cells. Three replicates were used. The results are presented as the mean ± SEM. ns, no significance; **$p < 0.01$, ****$p < 0.0001$, unpaired Student's t-test, two-sided.

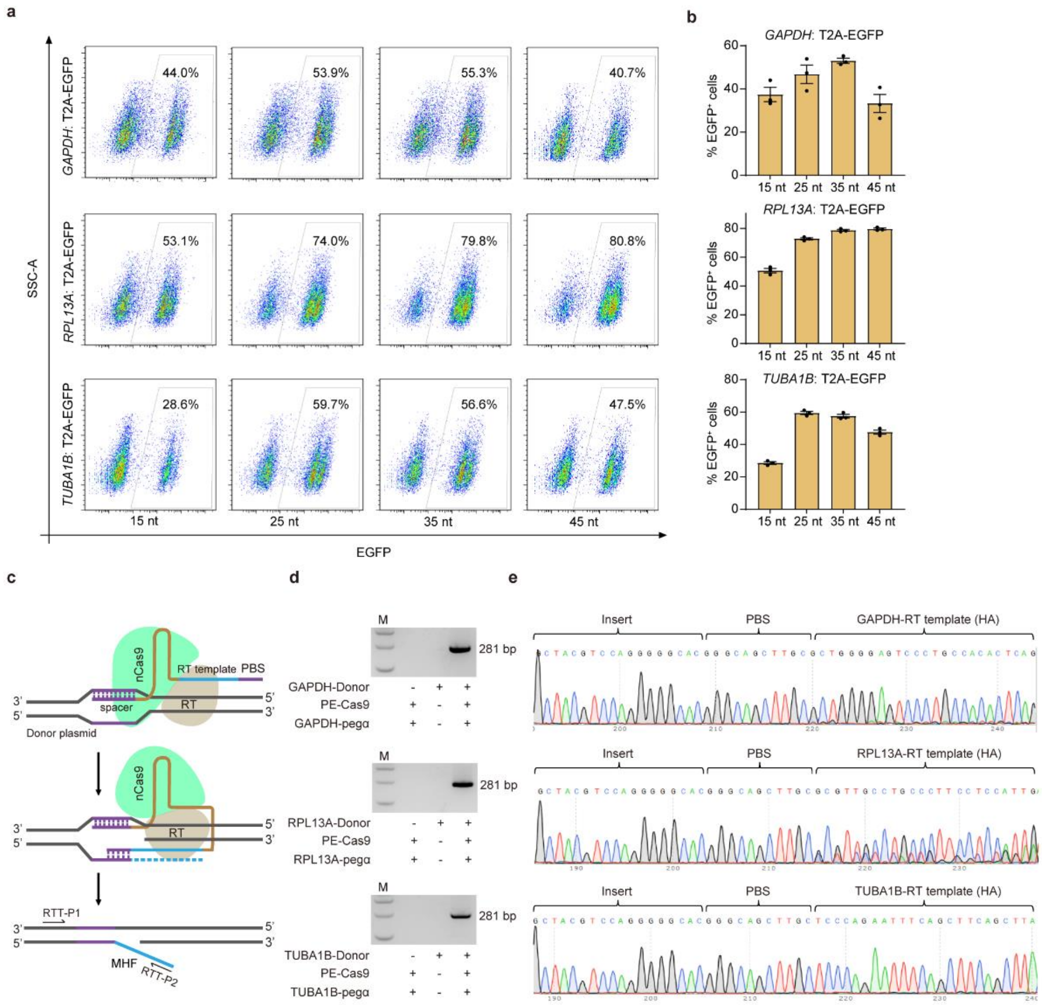

Supplementary Fig. S10 Optimization of RT template lengths of pegRNAs in PAINT 3.0-mediated transgene integration. a Scatter plots show editing efficiencies of PAINT 3.0-mediated T2A-EGFP integration at the 3'-UTR of *GAPDH*, *RPL13A*, and *TUBA1B* with varying pegRNA RT-template lengths. **b** Bar charts show statistics of the editing efficiencies of PAINT 3.0-mediated T2A-EGFP integration at the 3'-UTR of *GAPDH*, *RPL13A*, and *TUBA1B* with varying pegRNA RT-template lengths. PegRNAs with 35-nt RT templates show highest editing efficiencies in most cases. **c** Diagram shows the detection primed donors with a PCR-based method. The prime editor (PE) generates a single-stranded MH (MHF) at the nick site, which can be amplified by PCR with specific primers. **d** Identification of the primed single-

stranded MHs by PCR. Primers RTT-P1/GAPDH-RTT-P2, RTT-P1/RPL13A-RTT-P2, and RTT-P1/TUBA1B-RTT-P2 amplify PCR products of a specific size (281 bp) on PE-primed donors. **e** Sanger sequencing of the PCR products confirmed pegRNA-templated elongation of single-stranded MHs on the nicked DNA strands of PAINT 3.0-donors.

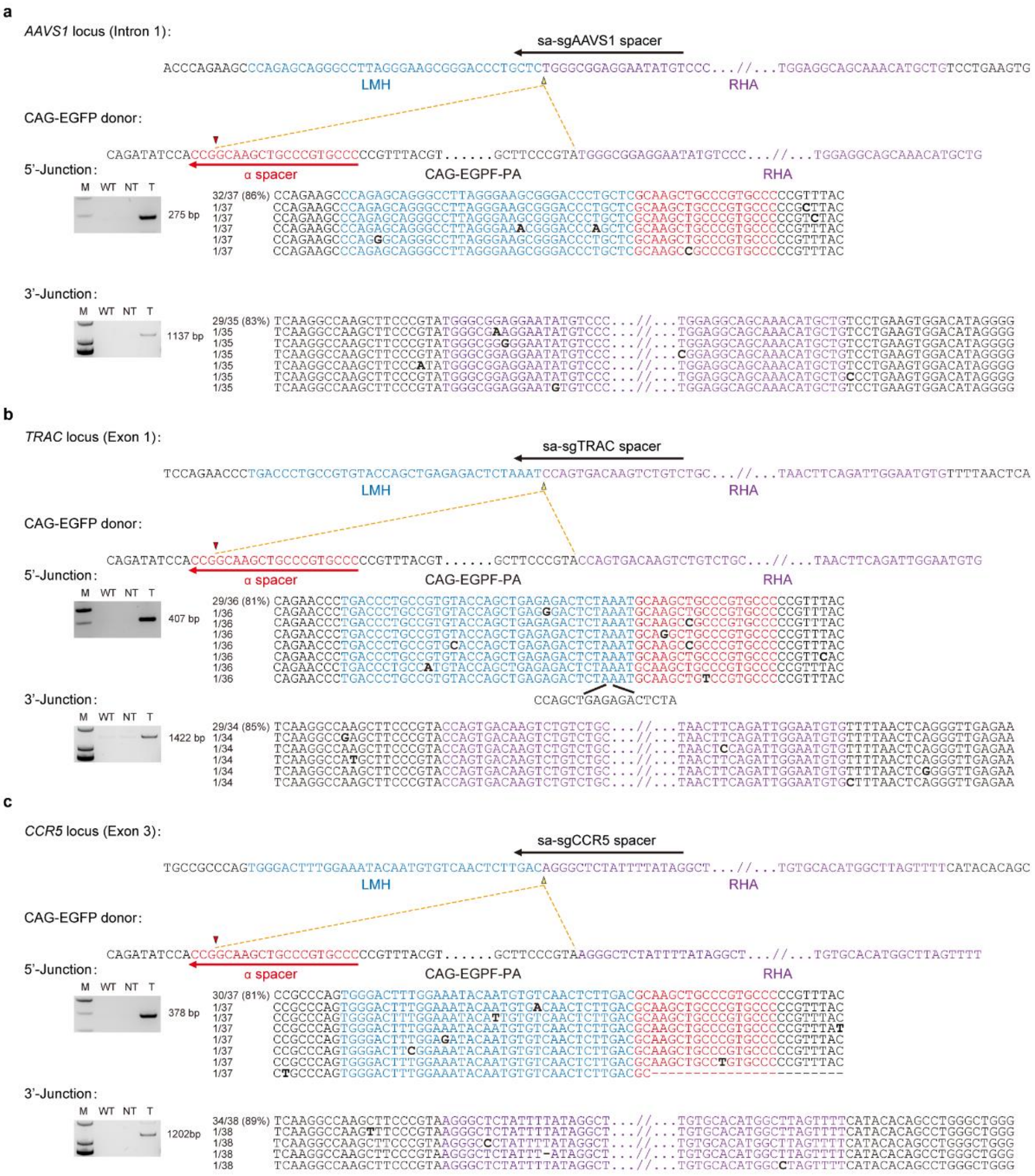
a
AAVS1 locus (Intron 1):
sa-sgAAVS1 spacer
LMH
RHA
CAG-EGFP donor:
5'-Junction:
α spacer
CAG-EGPF-PA
RHA
M WT NT T
275 bp
3'-Junction:
1137 bp
b
TRAC locus (Exon 1):
sa-sgTRAC spacer
LMH
RHA
CAG-EGFP donor:
5'-Junction:
α spacer
CAG-EGPF-PA
RHA
407 bp
3'-Junction:
1422 bp
c
CCR5 locus (Exon 3):
sa-sgCCR5 spacer
LMH
RHA
CAG-EGFP donor:
5'-Junction:
α spacer
CAG-EGPF-PA
RHA
378 bp
3'-Junction:
1202bp

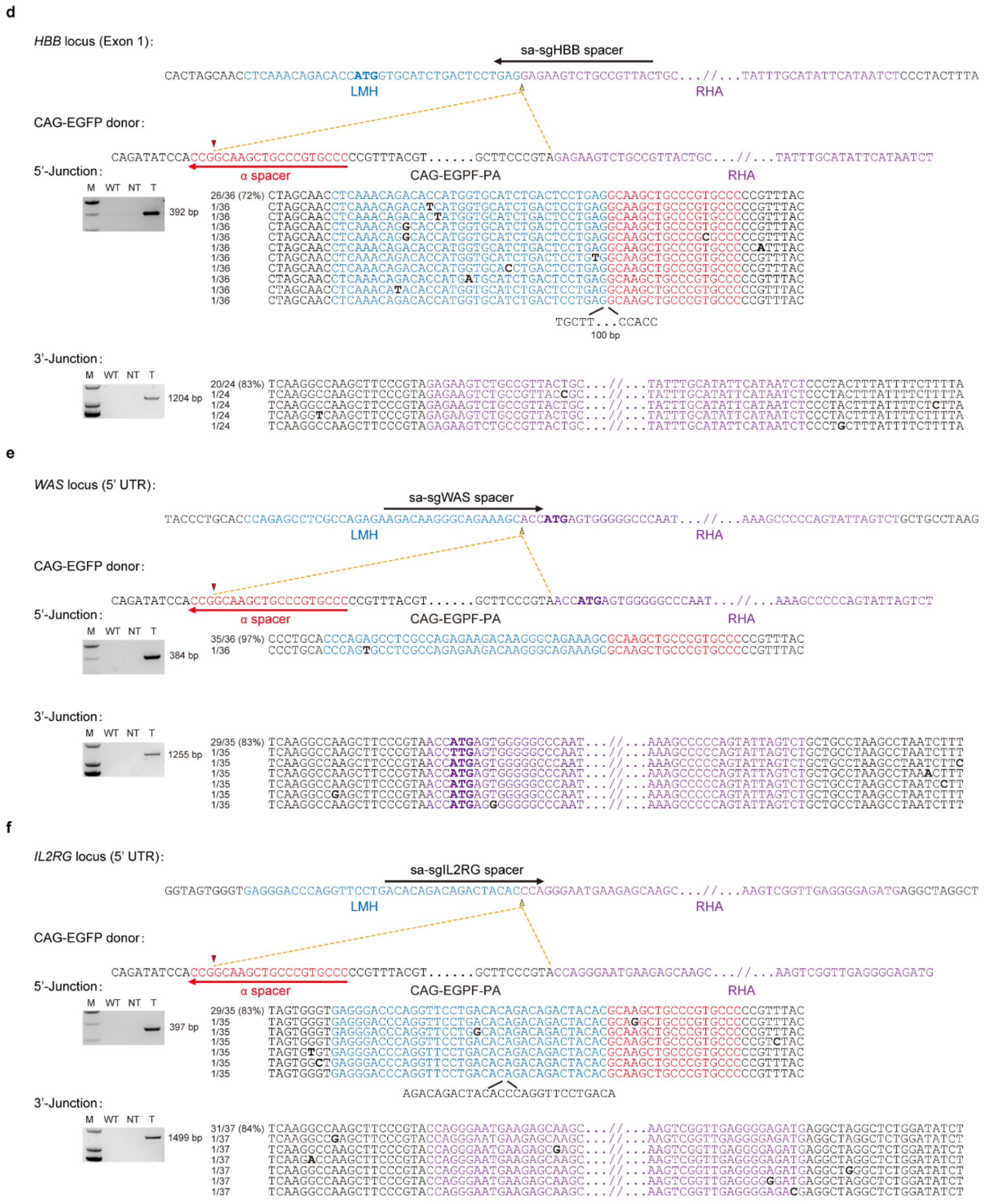

Supplementary Fig. S11 PAINT 3.0-mediated CAG-EGFP targeted integrations at therapeutically relative loci. a Genotyping and Sanger sequencing of PAINT 3.0-mediated CAG-EGFP targeted integration at Intron 1 of human *AAVS1* gene in K562 cells. Primers AAVS1-P1/AAVS1-P2 and AAVS1-P3/AAVS1-P4 amplify the 5' junction (275 bp) and 3' junction (1137 bp) on correctly edited *AAVS1* alleles, respectively. **b** Genotyping and Sanger

sequencing of PAINT 3.0-mediated CAG-EGFP targeted integration at Exon 1 of human *TRAC* gene in K562 cells. Primers TRAC-P1/TRAC-P2 and TRAC-P3/TRAC-P4 amplify the 5' junction (407 bp) and 3' junction (1422 bp) on correctly edited *TRAC* alleles, respectively. **c** Genotyping and Sanger sequencing of PAINT 3.0-mediated CAG-EGFP targeted integration at Exon 3 of human *CCR5* gene in K562 cells. Primers CCR5-P1/CCR5-P2 and CCR5-P3/CCR5-P4 amplify the 5' junction (378 bp) and 3' junction (1202 bp) on correctly edited *CCR5* alleles, respectively. **d** Genotyping and Sanger sequencing of PAINT 3.0-mediated CAG-EGFP targeted integration at Exon 1 of human *HBB* gene in K562 cells. The target site is near the translation start site and covers most of the mutations causing thalassemia. Primers HBB-P1/HBB-P2 and HBB-P3/HBB-P4 amplify the 5' junction (392 bp) and 3' junction (1204 bp) on correctly edited *HBB* alleles, respectively. **e** Genotyping and Sanger sequencing of PAINT 3.0-mediated CAG-EGFP targeted integration at the 5'-UTR of human *WAS* gene in K562 cells. The target site covers all reported mutations causing X-linked thrombocytopenia. Primers WAS-P1/WAS-P2 and WAS-P3/WAS-P4 amplify the 5' junction (384 bp) and 3' junction (1255 bp) on correctly edited *WAS* alleles, respectively. **f** Genotyping and Sanger sequencing of PAINT 3.0-mediated CAG-EGFP targeted integration at the 5'-UTR of human *IL2RG* gene in K562 cells. The target site covers all reported mutations causing X-linked severe combined immunodeficiency (X-SCID). Primers IL2RG-P1/IL2RG-P2 and IL2RG-P3/IL2RG-P4 amplify the 5' junction (397 bp) and 3' junction (1499 bp) on correctly edited *IL2RG* alleles, respectively.

Triangles indicate the cleavage sites in the genomic DNA and exogenous donors. LMH, left micro-homologue; RHA, right homologous arm; NT, non-target; T, on-target.

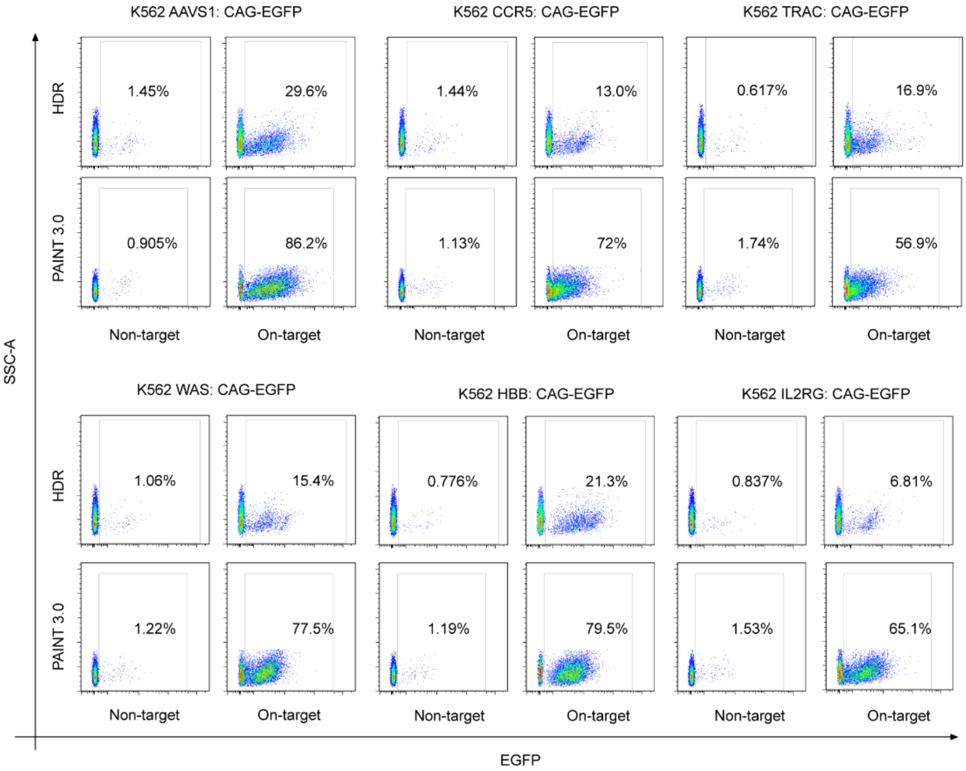

Supplementary Fig. S12 FACS scatter plots showing PAINT 3.0-mediated CAG-EGFP targeted integration at therapeutically relevant genomic loci in K562 cells.

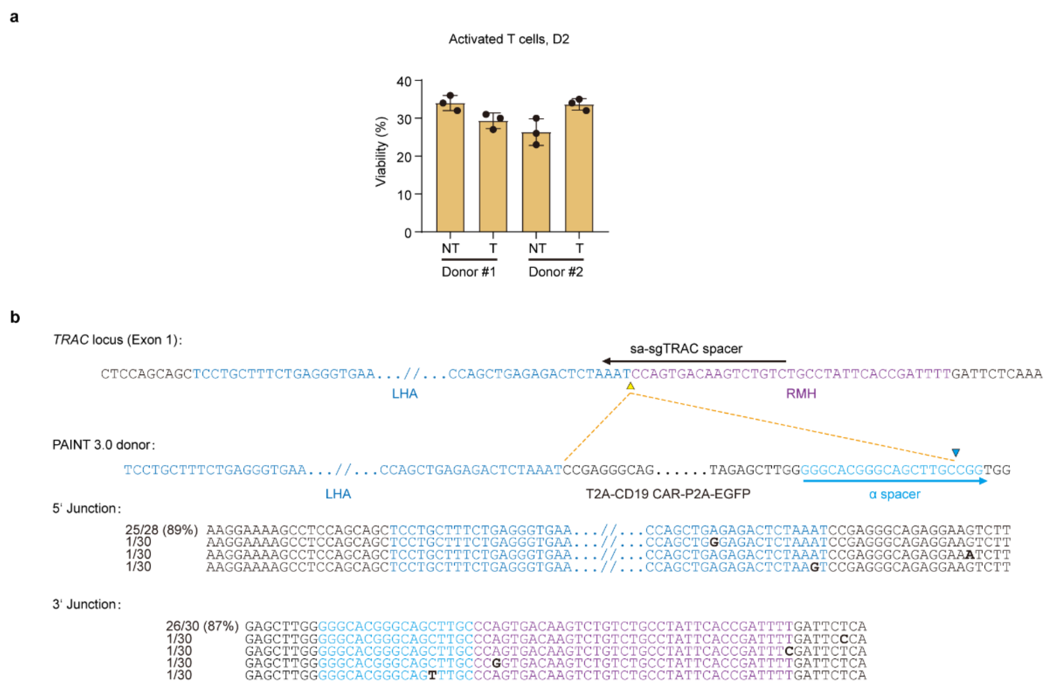

Supplementary Fig. S13 PAINT 3.0 mediates high-efficiency non-viral genome editing in primary T cells. a Viability of primary T cells after plasmids electroporation. Live T cells were counted for three times by Trypan Blue staining. NT, non-target; T, on-target. **b** Sanger sequencing confirms on-target integration of the CD19 CAR-EGFP transgene cassette in primary T cells.

Legends of Supplementary Tables

Supplementary Table S1: Sequences of generic and gene-specific spacers targeted by CRISPR/Cas systems.

Supplementary Table S2: Sequences of transgenes for targeted integration.

Supplementary Table S3: Sequences of genotyping primers, ddPCR primers and ddPCR probes.
